# Potential for and Distribution of Enzymatic Biodegradation of Polystyrene by Environmental Microorganisms

**DOI:** 10.3390/ma14030503

**Published:** 2021-01-21

**Authors:** Liyuan Hou, Erica L.-W. Majumder

**Affiliations:** Department of Chemistry, SUNY College of Environmental Science and Forestry, Syracuse, NY 13210, USA; lhou02@esf.edu

**Keywords:** plastics, polystyrene biodegradation, enzymatic biodegradation, monooxygenase, alkane hydroxylase, cytochrome P450

## Abstract

Polystyrene (PS) is one of the main polymer types of plastic wastes and is known to be resistant to biodegradation, resulting in PS waste persistence in the environment. Although previous studies have reported that some microorganisms can degrade PS, enzymes and mechanisms of microorganism PS biodegradation are still unknown. In this study, we summarized microbial species that have been identified to degrade PS. By screening the available genome information of microorganisms that have been reported to degrade PS for enzymes with functional potential to depolymerize PS, we predicted target PS-degrading enzymes. We found that cytochrome P4500s, alkane hydroxylases and monooxygenases ranked as the top potential enzyme classes that can degrade PS since they can break C–C bonds. Ring-hydroxylating dioxygenases may be able to break the side-chain of PS and oxidize the aromatic ring compounds generated from the decomposition of PS. These target enzymes were distributed in Proteobacteria, Actinobacteria, Bacteroidetes, and Firmicutes, suggesting a broad potential for PS biodegradation in various earth environments and microbiomes. Our results provide insight into the enzymatic degradation of PS and suggestions for realizing the biodegradation of this recalcitrant plastic.

## 1. Introduction

Over 300 million tons of plastics were produced worldwide every year [1], only 21% of which has been recycled or incinerated, and the rest of plastic wastes are released into the environment [2]. Plastic waste undergoes gradual fragmentation into microplastics (MPs) or nanoplastics (NPs) through weathering, photolysis, abrasion, mechanical, and microbial decomposition, resulting in the ubiquity and persistence of plastic fragments in the environment [3]. Moreover, these plastic wastes could harm the environment and living creatures in many ways, including disturbing the food web, accumulation in animals, entangling animals, enhanced toxicity with absorbing contaminants, carrying and transferring harmful algae and pathogens [4]. There is increasing evidence that various plastics can be degraded through microbial-mediated biodegradation. Certain plastic-degrading microorganisms including bacteria and fungi were found in various environments such as marine MPs-associated biofilms [5], landfills [6], wastewater treatment plants [7], compost [8], guts of mealworms [9], mangrove sediment [10], etc. While these studies reported the microbial consortium that can degrade synthetic polymers, they did not identify the microbial strains primarily responsible for the biodegradation of plastics [6,9,11]. Despite this, some researchers have been able to develop engineered microorganisms [11,12] and extract enzymes [12,13] for synthetic polymer degradation. Although an increasing number of microorganisms have been isolated and identified recently, the responsible enzymes or associated degradation pathways are rarely identified for many types of synthetic polymers.

In terms of biodegradability, plastics can be classified into biodegradable and non-biodegradable plastics. The biodegradable polymers with functional groups, such as ester, amide, and urethane, or polymers with carbon backbones, can be hydrolyzed via microorganisms [14]. For instance, polyhydroxyalkanoates (PHA) and polylactic acid (PLA), are bio-based biodegradable polyester and is produced in nature by bacterial fermentation [15]. Synthetic polymers including polyurethane (PUR), polyethylene (PE), polyamide (PA), polyethylene terephthalate (PET), polystyrene (PS), polyvinylchloride (PVC), and polypropylene (PP) require a long time to decompose [16]. Although ureases, esterases, and proteases were reported to act on the urethane and ester bonds of PUR, no enzymes have been defined that act on polyurethane ethers. Regarding PE, PA, PVC, and PP, specific enzymes that can degrade these polymers have not yet been identified to the gene level. Various enzymes of the hydrolase family, such as lipases, carboxylesterases, cutinases, and esterases have been shown to degrade PET to different extents [3]. PET hydrolase and mono-(2-hydroxyethyl) terephthalic acid (MHET) hydrolase from *Ideonella sakaiensis* can decompose PET into monomers (i.e., terephthalic acid and ethylene glycol) [17]. Additionally, Austin et al. narrowed the binding cleft through the mutation of two active-site residues to conserved amino acids in cutinases, which enhanced the enzyme’s ability in PET degradation [12].

As for PS, an aromatic thermoplastic with a C–C backbone, defined enzymes that act on PS were not yet reported. However, although the biodegradation rate is slow compared to organic matter [18], several microbial strains and consortium were able to degrade PS, using it as a sole carbon source. For instance, the brown-rot fungus *Gloeophyllum trabeum* caused the superficial oxidation of PS films [19]. *Bacillus cereus* and *Bacillus gottheilii* obtained from mangrove sediments reduced the weight of PS granules by 7.4% and 5.8%, respectively, within 40 days [10]. *Cupriavidus necator* H16 converted PS into biodegradable polyhydroxyalkanoates [20]. Both *Pseudomonas* spp. and *Bacillus* spp. showed a degradation ability in brominated high impact PS [21]. Despite the identification of microbes with the PS biodegradation capability, the mechanism of PS degradation has not been studied nor the identification of the responsible enzymes and biodegradation pathway.

Some researchers have proposed potential PS degrading enzymes, such as lipases, esterases, and oxidative enzymes [22,23]. Since the stability of C–C bonds decreases under alkaline conditions, alkane hydroxylases may break the PS main-chain C–C bonds under acidic or alkaline conditions [24]. However, accommodating bulky styrene groups in the active site may hinder the enzymatic breakage of C–C bonds. Przemieniecki S.W. et al. (2020) reported that β-Galactosidase, acid phosphatase, β-glucuronidase, naphthol-AS-BI-phosphohydrolase, leucine arylamidase, and alkaline phosphatase showed higher activities in the gut bacterial community of mealworms (*Tenebrio molitor*) [22]. Thus, the presence of known depolymerase enzymes classes that act on long-chain alkane groups or polymers with aromatic residues suggests that the PS depolymerase enzyme activity may exist in nature [25]. Remaining major hurdles for effective PS biodegradation are identifying the enzymes with the depolymerization functional capability at the gene, amino acid, or DNA sequence level and determining the involved degradation reactions and mechanisms. Furthermore, the PS depolymerization product is its monomer styrene, which is readily biodegraded into tricarboxylic acid cycle precursors by the styrene catabolism pathways in many microorganisms including *Pseudomonas*, *Rhodococcus*, *Xanthobacter*, and *Nocardia* [16]. Styrene is aerobically degraded through two main pathways, one attacking the vinyl group and the other based on direct ring cleavage [26]. Under anaerobic conditions, styrene is converted into phenylacetic acid (PAA) [26] and then to benzoic acid [27]. This makes it possible to completely biodegrade PS, if the depolymerization enzyme can be identified and optimized.

In this study, we aim to (1) summarize microbial species that have been identified to degrade PS; (2) predict enzymes that potentially have the ability to depolymerize PS from the genomes of known PS-degraders; (3) analyze the distribution of these enzyme targets in microbiomes of different environments that experience plastic contamination; and (4) predict the enzymatic reactions involved the PS biodegradation processes.

## 2. Materials and Methods

### 2.1. Literature Search for Microbes and Enzymes with Potential PS Degrading Ability

**Figure 1:**
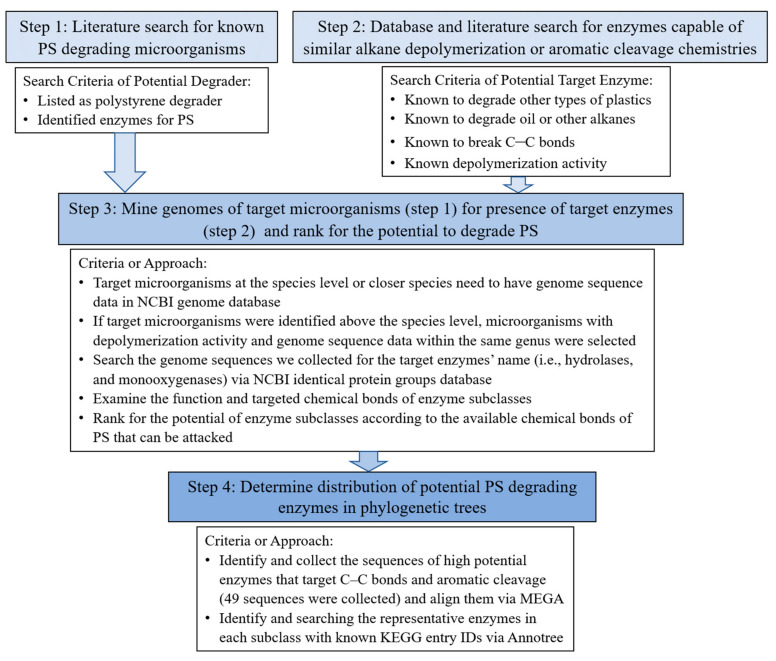
Identification of potential polystyrene (PS) degrading microbes and enzymes strategy.

As shown in Figure 1, the research strategy consisted of a systematic review of the literature and genome mining. The first step (step 1) included finding publications between 2010 to 2020 that investigated the strains or enzymes that degrade PS using ‘microbial degradation’, ‘enzymes’, and ‘PS’ as the keywords in databases, such as Scopus, PubMed, Science Direct, Journal Storage (JSTOR), etc. Literature relating to the isolation of PS degrading species, the process of microbial degradation of PS, and the potential enzymes involved were selected for further investigation and are reported in Table 1. The next step (step 2) comprised of identifying publications that described enzymes or microbes with known functions for conducting similar chemistries as to what would be required to depolymerize PS including: Degraders for other types of plastic and oil, enzymes that break C–C bonds or aromatic rings, and depolymerization enzymes.

### 2.2. Mining Genomes of Target Microorganisms for the Presence of Target Enzymes

Based on species-level identification and availability of a sequenced genome of the target microorganism or a close relative, we narrowed down the list of potential PS degrading microorganisms (step 3) in Table 1 to those reported in Table 2. We collected the genome sequence data of target microorganisms at the species level from the NCBI genome database. If the genome sequence data was missing, then genome sequences of the closest-related species with a sequenced genome were collected in their place. For microorganisms that had only been identified in the literature at higher taxonomic levels (e.g., genus, family, or above), other microorganisms from the same genus with both known depolymerization activity (e.g., other types of plastic degraders and oil degraders) and genome sequences were selected as the representative strain. Major enzyme classes that may be involved in PS degradation such as hydrolases and monooxygenases were used as the keywords to search in the NCBI ‘identical protein groups’ database within the targeted microorganisms in Table 2 (Figure 1). InterPro was used to determine the type of chemical bonds each enzyme subclasses acts upon. We then ranked the potential of enzyme subclasses towards the ability of PS depolymerization according to the availability of chemical bonds in PS that could be attacked by each type of enzyme.

### 2.3. Visualization of Enzyme Sequence Relationships and Distributions in Genomes and Phylogentic Trees

Different subclasses of hydrolases and monooxygenases were further examined along with enzymes that act on the C–C bonds and aromatic rings or catalyze oxidation reactions that were selected as the high potential enzymes from the target microorganisms listed in Table 2 (step 4). A total of 49 DNA sequences of high potential enzymes were collected through the NCBI’s ‘identical protein groups’ database. Sequences were then aligned with the Multiple Sequence Comparison by Log-Expectation (MUSCLE) algorithm in Molecular Evolutionary Genetics Analysis (MEGA) X program to detect the potential relationship among these enzymes [40]. The neighbor-joining method in MEGA v6 was used to construct phylogenetic trees to visualize the clustering of the DNA sequences [41,42]. A precomputed and visualized genome-wide phylogeny with taxonomic annotations showing the representative enzymes with known Kyoto Encyclopedia of Genes and Genomes (KEGG) entry IDs in each subclass in domain bacteria was downloaded from AnnoTree. Representative enzymes in subclass enzymes (e.g., monooxygenases, hydroxylases, and aromatic-ring-hydroxylating dioxygenase) included alkane 1-monooxygenase (K00496), 4-hydroxybenzoate 3-monooxygenase (K00481), 3-hydroxybenzoate 6-monooxygenase (K22270), 2-octaprenyl-6-methoxyphenol hydroxylase (K03185), 2-octaprenyl-3-methyl-6-methoxy-1,4-benzoquinol hydroxylase (K03184), and aromatic-ring-hydroxylating dioxygenase (K05709). Phylogenetic distributions of the different bacterial genomes containing enzymes that were involved in the side-chain oxygenation of styrene (i.e., styrene monooxygenase (K14481), styrene monooxygenase reductase (K14482), styrene-oxide isomerase (K18312), and phenylacetaldehyde dehydrogenase (K00146)), and direct ring cleavage of styrene (i.e., cis-2,3-dihydrobiphenyl-2,3-diol dehydrogenase (K08690) and catechol 2,3-dioxygenase (K00446)) [43] were also positioned within the tree generated by Annotree, respectively.

## 3. Results and Discussion

### 3.1. Potential PS Degradting Enzymes

A total of 21 species were screened and selected, which may exhibit the ability to break PS with the presence of multiple functional enzymes (Table 2). A wide range of hydrolase and monooxygenase subclasses was included. For example, hydrolases included esterase, carboxylesterase, alpha/beta hydrolase, etc. Monooxygenases were comprised of oxidoreductase, alkane 1-monooxygenase, cytochrome P450 alkane hydroxylase, etc. (Table S1).

**Figure 2:**
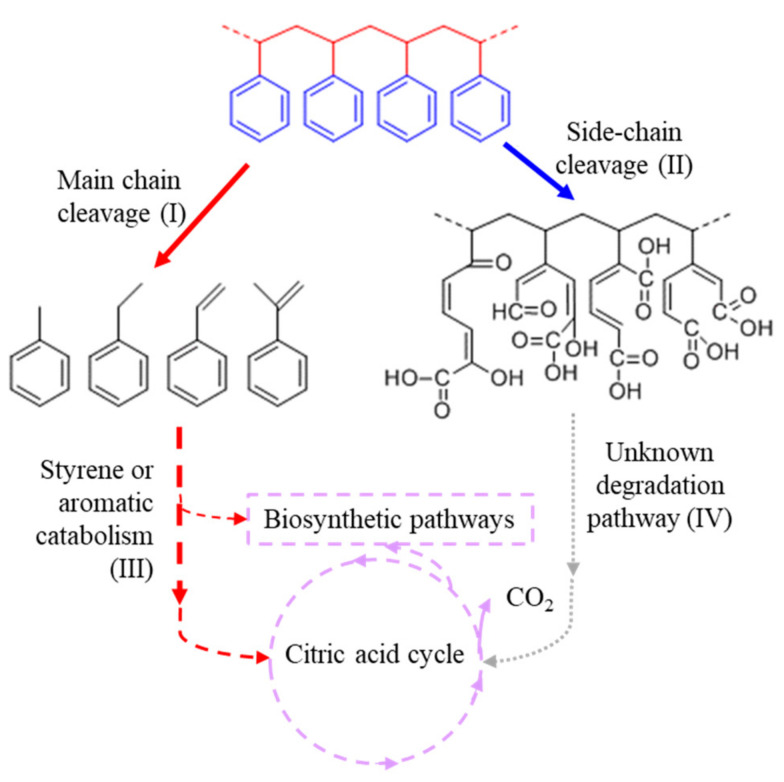
Proposed biodegradation pathway of PS. The pathway involves several steps including main chain cleavage (I) followed by styrene or aromatic catabolism (III), and side-chain cleavage (II) followed by unknown degradation pathway (IV) that break the PS polymer into smaller compounds that can be metabolized in microorganisms. The red indicates the pathway for main chain cleavage while blue indicates the pathway for side-chain cleavage. Potential aromatic ring cleavages on PS side-chain are presented within the same PS main chain.

The surface attachment of microorganisms to the polymer is an essential step for the biodegradation of PS. As microbes attach to surfaces, they colonize the area and form a biofilm while secreting extracellular polymeric substances and proteins. The depolymerase enzymes can be excreted to the extracellular milieu, which in combination with the other excreted biomaterials can act to convert hydrophobic substrates to have a more hydrophilic surface [32]. This, in turn, enhances the attachment of bacteria or other types of microorganisms and enzymes to their target substrates. For instance, PETase has a highly polar surface that favors binding to more hydrophilic regions of the substrate [44]. After this, polymer decomposition starts with the initial cleavage of insoluble macromolecules into smaller fragments that can then be taken up by microorganisms for further use. However, the extracellular and intracellular degradation of PS has not yet been evaluated explicitly. As a result, in this study, we propose the degradation pathway of PS involves several steps (Figure 2). Firstly, based on the chemical structure of PS, either the β-carbon of the chain (main chain cleavage) or the aromatic ring (side-chain cleavage) may be attacked by functional enzymes so that PS polymers can be broken down into smaller compounds. For the main chain cleavage, styrene and compounds that have analogous structures with styrene can be processed through the styrene or aromatic catabolism. As for the side-chain cleavage, there is a potential for some aromatic ring hydroxylases or monooxygenases to break the aromatic ring of PS as shown in Figure 2. The PS with ring cleavages could be further decomposed to short chain hydrocarbons through unknown degradation pathways. The products from styrene or aromatic metabolism and unknown degradation pathways following side-chain cleavage could then be processed through microbial energy metabolism and ultimately mineralized as CO_2_ through the citric acid cycle. They may also be exploited for the biosynthesis of valuable products through other metabolic pathways [20] (Figure 2).

Among all enzymes that have been examined (Table S1), alkane hydroxylase, monooxygenase, cytochrome P450, aromatic ring hydroxylase, and esterase/alpha/beta hydrolase were the main enzymes that were widely present in target microorganisms. Oxidoreductases including cytochrome P450, monooxygenase, alkane hydroxylase, and aromatic ring hydroxylase, exhibit a higher possibility to decompose PS when compared to hydroxylases such as esterase and alpha/beta hydrolase (Table 3). This is because PS does not have ester bonds. Esterase/alpha/beta hydrolase may play a less important role in the depolymerization steps. Esterases belonging to hydroxylases (EC 3.1.1) show broad substrate specificity toward oxo-esters or thio-esters of various fatty acids [23]. Alpha/beta hydrolases can function as hydrolases, lyases, transferases, hormone precursors or transporters, chaperones, or routers of other proteins [45]. The wide presence of these enzymes in the genomes of target microorganisms may indicate their role in the cleavage of ester bonds in small compounds that may be formed during the depolymerization of PS (Figure 2).

Alkane hydroxylase, monooxygenase, and cytochrome P450 primarily act on the β-carbon of the carbon chain and play an important role in the main-chain cleavage (Figure 2). They may also be important in the unknown degradation pathway (Figure 2) to further depolymerize the PS polymer. Cytochromes P450, a superfamily of enzymes containing heme cofactors, are widely distributed in all kingdoms of life. The most common oxidative reaction catalyzed by cytochrome P450 is the monooxygenation of a substrate. Cytochrome P450 can participate in monooxygenase, peroxidase, and peroxygenase reactions. For instance, Cytochrome P450 CPY152A1 (from *Bacillus subtilis*) in the presence of hydrogen peroxide can catalyze the hydroxylation of ethylbenzene and the epoxidation of styrene [46]. Similarly, cytochrome P450 CPY152B1 (from *Sphingomonas paucimobilis*) also catalyzed the epoxidation of styrene in the presence of hydrogen peroxide [47]. Cytochrome P450 CPY153s from *Alcanivorax borkumensis* and *Sphingomonas* sp. can act on long-chain alkanes [48,49]. Cytochrome P450 CYP116B5 from *Acinetobacter radioresistens* S13 can oxidize medium- (C14 and C16) and long- (C24 and C36) chain alkanes [50]. This initial oxidation can be governed by alkane-degrading cytochromes P450 to convert alkane into a primary alcohol, which is then further oxidized to the corresponding aldehyde, and finally converted into a fatty acid [51]. Subterminal oxidation can also occur in some microorganisms and the corresponding secondary alcohols are oxidized to ketones, and then, a Baeyer–Villiger monooxygenase converts them to esters, which are subsequently cleaved to give rise to a primary alcohol and a common fatty acid [52]. As a result, cytochromes P450 may not only be able to break the C-C bonds resulting in the formation of single-ring aromatic compounds but also oxidize these single-ring aromatic compounds.

AlkB (alpha-ketoglutarate-dependent hydroxylase) related to alkane hydroxylases, which are normally non-heme iron monooxygenases, may also break the PS polymer chain. It was suggested that this type of enzyme contains a deep hydrophobic pocket formed by six transmembrane helices, and that the alkane substrate molecule slides into the pocket until the terminal methyl group is correctly positioned with the catalytic histidine residues, rendering a formation of an alcohol [51]. *Pseudomonas putida* GPo1 AlkB alkane hydroxylase oxidizes C_3_–C_13_ or C_10_–C_20_ n- n-alkanes [53]. Two AlkB type alkane hydroxylases that are related to AlkB from *Pseudomonas putida* GPo1, named AlkMa and AlkMb, also played important roles for *Acinetobacter* sp. M1 to grow on C_13_–C_44_ n-alkanes [54].

Monooxygenases that incorporate one atom of oxygen into the substrate may also contribute to the decomposition of PS. For instance, the alkane monooxygenase LadA from *Geobacillus thermodenitrificans* NG80-2 acts on long-chain alkanes (C_15_–C_36_) [55]. A Rieske-type monooxygenase from the *Pusillimonas* sp. strain T7-7 is able to oxidize C_5_–C_30_ alkanes [56]. Flavin-binding monooxygenases (AlmA) are involved in long-chain (C_20_–>C_32_) alkane degradation in bacteria of the *Acinetobacter* and *Alcanivorax* genera [57]. These enzymes, capable of alkane degradation, have the potential to be involved in the cleavage of the PS main chain, which is a substituted alkane. Furthermore, monooxygenases such as 4-hydroxybenzoate 3-monooxygenase and 3-hydroxybenzoate 6-monooxygenase may contribute to the oxidization of ring aromatic compounds (e.g., 4-hydroxybenzoate and 3-hydroxybenzoate) generated from the decomposition of PS main chain. These enzymes have also been found in microorganisms that degrade polycyclic aromatic hydrocarbons [58] and petroleum [59].

The main chain of PS is more likely to be cleaved compared to the aromatic ring substituent since the alkane chain C–H (410 KJ/mol) and C–C bonds (350 KJ/mol) are weaker and more susceptible to cleavage than the aromatic C=C bonds (680 KJ/mol) [60]. However, there is potential for aromatic ring hydroxylases to cause a preferential cleavage of the styrene group aromatic ring by incorporating two atoms of dioxygen into that styrene aromatic ring (Figure 2, pathway II). Thus, aromatic ring hydroxylases play a moderate role in the styrene or aromatic catabolism and the side-chain cleavage pathways. A ring-hydroxylating dioxygenase from *Rhodococcus* sp. P14 is capable of anthracene and benz[a]anthracene oxidization [61]. The ring-hydroxylating dioxygenase from *Sphingobium* sp. FB3 can also oxidize the benz[a]anthracene [62]. These ring-hydroxylating dioxygenases may also contribute to the oxidization of ring aromatic compounds after the decomposition of PS. For instance, biphenyl dioxygenase was essential for the growth of *Rhodococcus jostii* RHA1 on styrene [63]. Benzoate 1,2-dioxygenase from *Microbacterium esteraromaticum* SBS1-7 catalyzes benzoate catabolism, which has a similar chemical structure to the PS monomer product 2-hydro-1,2-dihydroxybenzoate [64].

**Figure 3:**
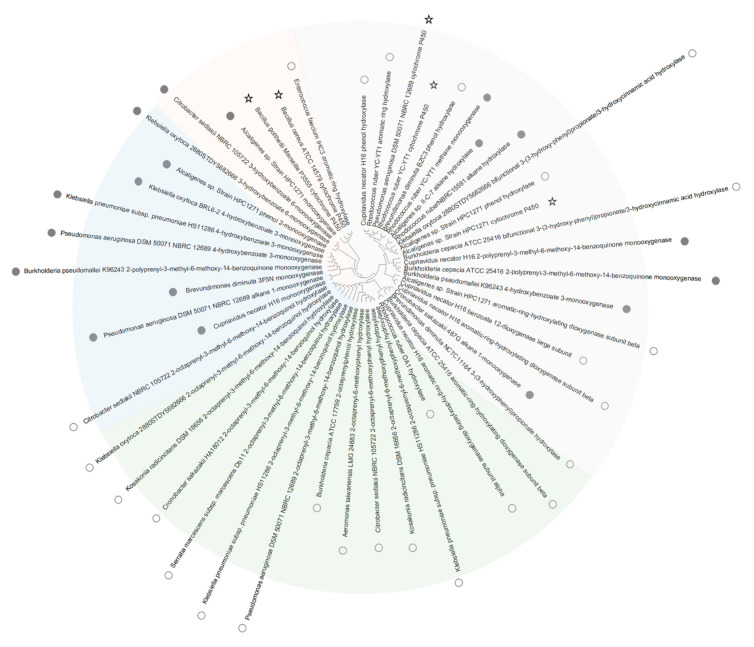
Phylogenetic tree of DNA sequences of higher depolymerization functional potential enzymes from target microorganisms listed in Table 2, including cytochrome P450 (star), monooxygenase (solid circle), and aromatic ring hydroxylase (hollow circle). Sectors of the tree in different colors indicate major enzyme subclasses. The evolutionary history was inferred using the Neighbor-Joining method [41]. The optimal tree with a sum of branch length = 23.57786440 is shown. The evolutionary distances were computed using the Maximum Composite Likelihood method [42] and are in the units of the number of base substitutions per site. The proportion of sites where at least 1 unambiguous base is present in at least 1 sequence for each descendent clade is shown next to each internal node in the tree. This analysis involved 49 nucleotide sequences as shown in Table S2. All ambiguous positions were removed for each sequence pair (pairwise deletion option). There were a total of 2530 positions in the final dataset.

By constructing a phylogenetic tree of higher potential subclass enzymes, we observed that some enzymes clustering together were from the same species (Figure 3). For instance, the phenol hydroxylase and cytochrome P450 of *Alcaligenes* sp. strain HPC1271 clustered. This indicated that some species may have several types of PS decomposition enzymes that have the same functions (Figure 3). A previous study demonstrated that *Acinetobacter* sp. DSM 17874 contains at least three n-alkane-oxidizing enzymes, which are involved in the oxidation of n-alkanes of different size ranges [51]. Additionally, some cytochrome P450 enzymes of certain species were grouped closer to aromatic ring hydroxylases (Figure 3). For instance, the cytochrome P450 of *Bacillus cereus* ATCC was closer to the aromatic ring hydroxylase of *Enterococcus faecium* IHC3 compared with the cytochrome P450 of *Bacillus gottheilii Marseille* P3555. Similarly, benzo(a)pyrene was degraded through catalysis by cytochrome P450 hydroxylase from *Bacillus thuringiensis* [65]. It was also observed that some cytochromes P450 and alkane monooxygenases clustered with some esterases (Figure S1), indicating their similar enzymatic structure. This may explain the statements made by previous studies that esterases were the functional enzymes in PS degradation since they showed high activities in those experiments [22].

It is hard to predict the optimal temperature and pH ranges, active sites, and cofactors for enzymatic degradation of PS because of the limited number of studies conducted related to this topic. However, monooxygenases LadA were active at temperatures ranging from 40 to 90 °C and at pH values from 6.0 to 8.8, which is promising for industrial application [66]. Cytochromes P450 normally require heme as the cofactor but some monooxygenases such as AlmA and LadA use flavin as the cofactor [51]. Fe^2+^ can enhance the activity of AlkB-related alkane hydroxylases, as they are iron dependent [53].

Understanding the degradation kinetics of enzymes can help to predict the degradation mechanism and the enzyme activity performance with PS as a substrate [67]. To date, few studies have been done to investigate the degradation kinetics of PS degrading enzymes. The degradation kinetics of PS without significant genome or protein engineering is likely to be slow compared to the biodegradation of organic matter, as is seen in other even highly biodegradable plastics which have long decomposition times. The heme-containing cytochrome P450 BM3 has high turnover numbers of up to 285 s^−1^ but has rather low regioselectivity for long-chain fatty acids [68]. Alkane monooxygenase, AlkBGT, from recombinant *Escherichia coli* W3110 (Pbt10) showed high conversion rates (104 U/g_CDW_) of medium chain-length fatty acid methyl esters [69]. By improving cytochrome P450 monooxygenases activities, the specific whole-cell activity of cytochrome P450 monooxygenases from *Pseudomonas taiwanensis* VLB120 pSEVA_Cyp had been improved from 34 to 55 U/g_CDW_ in oxidizing cyclohexane [70]. More research is needed in determining the reaction order, reaction rate, and enzyme deactivation rate in PS degrading processes, as overcoming this barrier will be key for practical applications of biodegradation of all plastics.

### 3.2. Distribution of Potential PS Degradting Enzymes across Domian Bacteria

**Figure 4:**
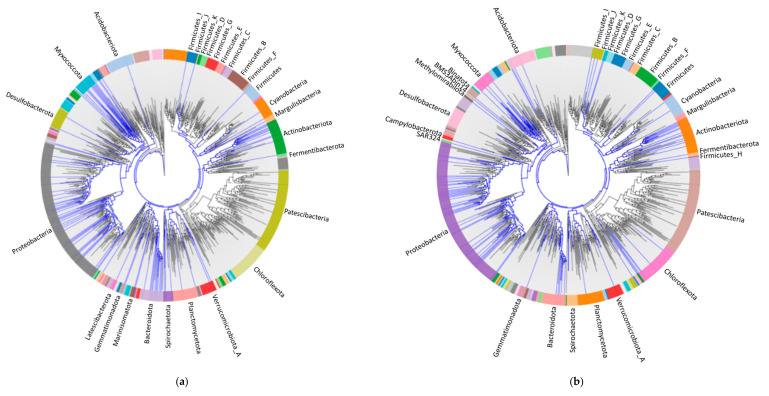

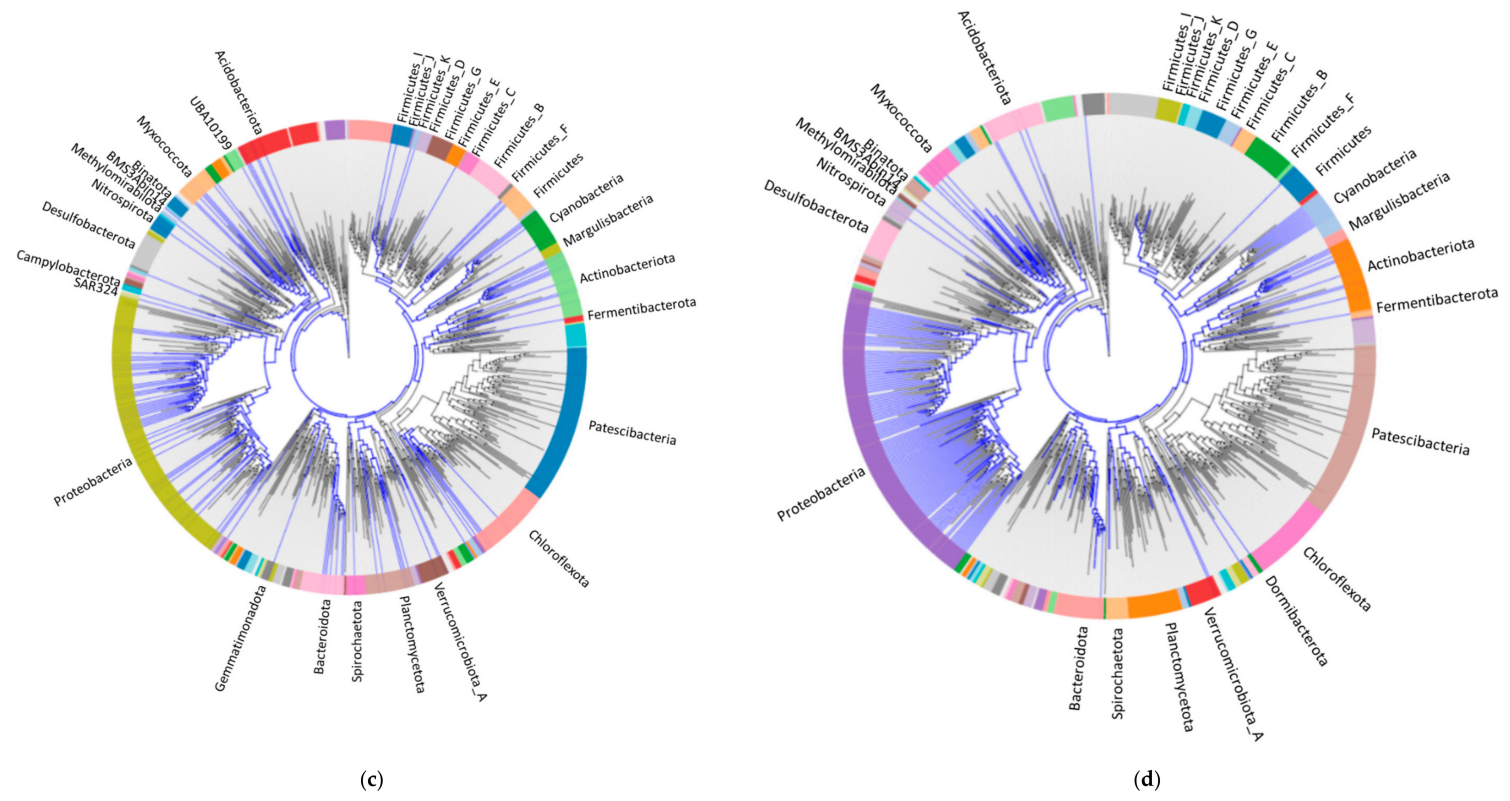

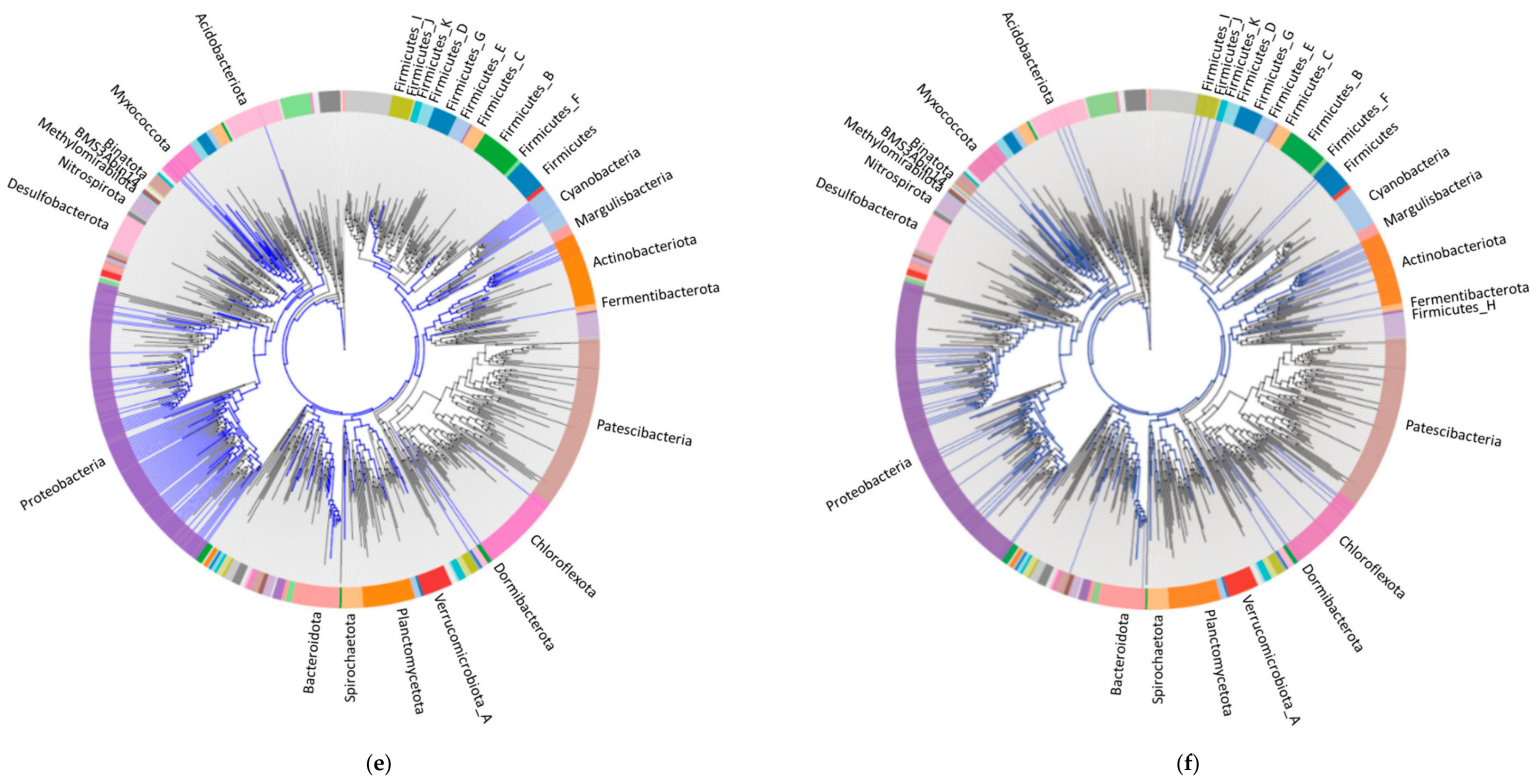

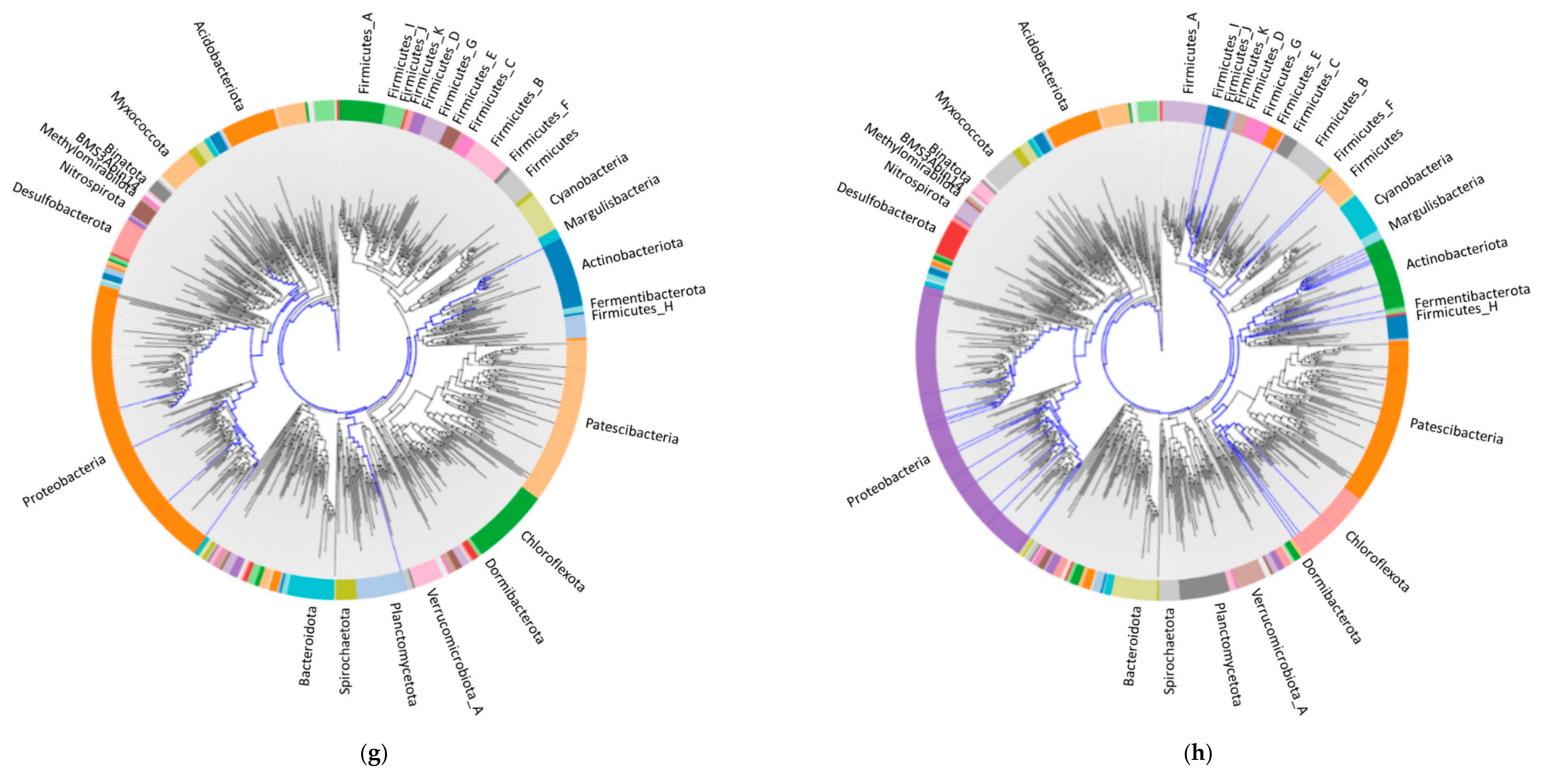
Visualization of phylogenetic distribution of high potential PS-degrading enzymes from AnnoTree. Enzymes depicted are alkane 1-monoxygenase (**a**), 4-hydroxybenzoate 3-monooxygenase (**b**), 3-hydroxybenzoate 6-monooxygenase (**c**), and 2-octaprenyl-6-methoxyphenol hydroxylase (**d**), 2-octaprenyl-3-methyl-6-methoxy-1, 4-benzoquinol hydroxylase (**e**), aromatic ring hydroxylating dioxygenase (**f**), enzymes involving in the side-chain oxygenation of styrene (**g**), and enzymes involving in direct ring cleavage of styrene (**h**). Blue branches in each phylogenetic tree represent the microbial species at the order level harboring the target high potential PS-degrading enzymes. Colored rings represent different phyla which these strains belong to. Due to the font size of phylogenetic trees, only the names of phyla with potential PS-degrading enzymes were presented.

The representatives of monooxygenase (i.e., alkane 1-monooxygenase, 4-hydroxybenzoate 3-monooxygenase, and 3-hydroxybenzoate 6-monooxygenase), hydroxylase (i.e., 2-octaprenyl-6-methoxyphenol hydroxylase, 2-octaprenyl-3-methyl-6-methoxy-1, and 4-benzoquinol hydroxylase), and aromatic ring hydroxylating dioxygenase, were further selected to investigate their distribution in the domain of bacteria in using AnnoTree. A total of 2724, 4122, and 3808 genome hits across domain of bacteria were observed for alkane 1-monooxygenase, 4-hydroxybenzoate 3-monooxygenase, and 3-hydroxybenzoate 6-monooxygenase, respectively. The main phyla that contain the alkane 1-monoxygenase were Proteobacteria (52.57% of total hits), Actinobacteria (26.65%), and Bacterioidota (15.05%) (Figure 4a). Similarly, Proteobacteria, Actinobacteria, and Bacterioidota were also the primary phyla for 4-hydroxybenzoate 3-monooxygenase (62.13%, 32.85% and 1.07% for Proteobacteria, Actinobacteria, and Bacterioidota, respectively) (Figure 4b) and 3-hydroxybenzoate 6-monooxygenase (50.63%, 39.81%, and 2.99% for Proteobacteria, Actinobacteria, and Bacterioidota, respectively) (Figure 4c). 2-octaprenyl-6-methoxyphenol hydroxylase (Figure 4d) and 2-octaprenyl-3-methyl-6-methoxy-1, 4-benzoquinol hydroxylase (Figure 4e) hit 8018 and 4247 genomes in three phyla of bacteria, which were Proteobacteria (>80%), Actinobacteria, and Cyanobacteria. Aromatic-ring-hydroxylating dioxygenase hit only 1159 genomes (Figure 4f), and Proteobacteria (50.04%), Actinobacteria (35.38%), and Firmicutes (6.82%) were the main phyla that may have aromatic ring hydroxylating dioxygenase. These results indicated that potential PS degrading enzymes were mainly distributed in Proteobacteria and Actinobacteria, and that Bacterioidota and Firmicutes may also have some potential functional enzymes. This result is consistent with the selected species listed in Table 2. Interestingly, many species in Proteobacteria, Bacterioidetes Actinobacteria, and Firmicutes were identified to be hydrocarbon-degrading bacteria in oil-contaminated environments [71]. Additionally, Proteobacteria and Actinobacteria were involved in polycyclic aromatic hydrocarbons pollution degradation and significantly decreased soil PHA contents [72]. Consistent with the results of target microorganisms for PS degradation listed in Table 2, *Burkholderia*, *Kosakonia*, *Pseudomonas*, and *Rhodococcus* are capable of degrading petroleum hydrocarbons [73]. Moreover, enzymes involving styrene degradation (Figure 4g,h) were also distributed in Proteobacteria and Actinobacteria, indicating that species in Proteobacteria and Actinobacteria have higher potential for PS degradation.

## 4. Conclusions

We predicted PS degrading enzymes with the highest potential for being capable of depolymerization, which included cytochromes P450, alkane hydroxylases, and monooxygenases. They may play a key role in breaking PS main-chain C–C bonds. Ring-hydroxylating dioxygenases may be able to break the side-chain of PS but may also contribute more to the oxidation of the aromatic compounds generated from the decomposition of PS. Within microorganisms, such high potential for depolymerizing PS enzymes were mainly distributed in Proteobacteria, Actinobacteria, Bacterioidota, and Firmicutes, indicating potential for plastic degradation in many earth environments. Additional work is needed to identify and investigate the activities of putative PS degrading enzymes in known PS degrading microbial species and the catalytic mechanisms of these enzymes for this chemical reaction.

## Figures and Tables

**Table 1:** Literature search for known PS degrading microorganisms and predicted enzymes.

Pure or Mixture Species	Microorganisms	Potential Enzymes	Reference
Pure species (two)	*Bacillus cereus* and *Bacillus gottheilii*	N/A ^1^	[10]
Pure species	*Cupriavidus necator* H16	N/A	[20]
Microbial consortium from superworms’ guts	*Alcaligenes* sp., *Pseudomonas* sp., or *Acinetobacter* sp., and *Klebsiella pneumoniae*	N/A	[28]
Microbial consortium from an open waste dump	*Shingobacterium* sp., *Flavobacterium* spp., *Pseudoxanthomonas* sp., *Burkholderia* sp., *Xanthobacter* sp., *Methylobacter* sp., *Methylococcus* sp., *Methylocella* sp., *Methylocystis* sp., *Nitrobacter hamburgensis*, *Nitrobacter wingogradskyi* and *Nitrosomonas* sp.	N/A	[6]
Pure species	*Penicillium variabile* CCF3219	N/A	[29]
Pure species (two)	*Pseudomonas* spp. and *Bacillus* spp.	Lipase and esterase	[21]
Pure species (four)	*Enterobacter* sp., *Citrobacter sedlakii*, *Alcaligenes* sp. and *Brevundimonas diminuta*	Extracellular deploymerase enzymes	[30]
Pure species(three)	*Serratia marcescens* PCM3034, *Klebsiella oxytoca* PCM3036 and *Pseudomonas aeruginosa* PCM3035.	N/A	[31]
Pure species	*Gloeophyllum trabeum* DSM 1398	Oxidative (exo)enzymes	[19]
Pure species	*Exiguobacterium* sp. YT2	N/A	[25]
Pure species from superworms’ guts	*Pseudomonas aeruginosa* strain DSM 50071	N/A	[32]
Microbial consortium from moths’ guts	*Enterococcus* sp., *Geobacillus* sp. *Serratia marcescens*, *Pseudomonas* sp. and *Bacillus cereus.*	N/A	[9]
Microbial consortium from seawater	N/A	Alkane 1-monooxygenase	[5]
Microbial consortium from mealworms’ guts	*Citrobacter* spp., *Kosakonia* spp., *Listeria* spp. and *Nitrospira defluvii*	β-Galactosidase, acid phosphatase, β-glucuronidase, naphthol-AS-BI-phosphohydrolase, leucine arylamidase, and alkaline phosphatase	[22]
Microbial consortium from mealworms’ guts	*Listeria* sp., *Nitrospira defluvii*, *Pedomicrobium* sp., *Aquihabitans* sp., unclassified *Xanthomonadaceae*, unclassified *Saprospiraceae*, and unclassified *Burkholeriales*	N/A	[33]
Microbial consortium from mealworms’ guts	*Spiroplasmataceae*, *Enterococcaceae*, and *Enterobacteriaceae*	N/A	[34]
Microbial consortium from superworms’ guts	N/A	N/A	[35]
N/A	N/A	P450 monooxygenases	[24]
Microbial consortium from brackish water	*Burkholderiales*	N/A	[36]
Pure species	*Azotobacter beijerinckii* HM121	Hydroquinone peroxidase	[23]
Enrichment culture	*Winogradskyella*, *Salinimicrobium*, *Vibrio, Photobacterium* and *Pseudomonas*	N/A	[37]
Pure species	*Rhodococcus ruber* C208	N/A	[38]
Pure species (two)	*Exiguobacterium sibiricum* strain DR11 and *Exiguobacterium undae* strain DR14	Hydrolyzing enzymes	[39]

^1^ N/A indicates the paper did not describe or discuss the potential PS-degrading enzymes.

**Table 2:** Identification of potential PS-degrading microorganisms with known genome sequences and potential enzymes within the genome.

Kingdom	Phylum	Family	Genus	Species	Number of Hydrolases in Search Result	Number of Monooxygenases in Search Result
Bacteria	Firmicutes	*Bacillaceae*	*Bacillus*	*Bacillus cereus* ATCC 14579	290	24
	Firmicutes	*Bacillaceae*	*Cytobacillus*	*Cytobacillus gottheilii* ASM163634v1	156	11
	Firmicutes	*Enterococcaceae*	*Enterococcus*	*Enterococcus faecium* DO	177	7
	Firmicutes	*Listeriaceae*	*Listeria*	*Listeria innocua* Clip11262	90	9
	Firmicutes	*Bacillales Family XII. Incertae Sedis*	*Exiguobacterium*	*Exiguobacterium sibiricum* 255-15	74	11
	Proteobacteria	*Burkholderiaceae*	*Cupriavidus*	*Cupriavidus necator* N-1	254	59
	Proteobacteria	*Burkholderiaceae*	*Burkholderia*	*Burkholderia cepacia* ATCC 25416	292	53
	Proteobacteria	*Burkholderiaceae*	*Burkholderia*	*Burkholderia pseudomallei* K96243	270	40
	Proteobacteria	*Enterobacteriaceae*	*Klebsiella*	*Klebsiella pneumoniae* subsp. *pneumoniae HS11286*	193	34
	Proteobacteria	*Enterobacteriaceae*	*Kosakonia*	*Kosakonia radicincitans* DSM 16656	132	19
	Proteobacteria	*Enterobacteriaceae*	*Cronobacter*	*Cronobacter sakazakii* ASM98282v1	2662 *	254 *
	Proteobacteria	*Enterobacteriaceae*	*Klebsiella*	*Klebsiella oxytoca* ASM102219v1	6807 *	801 *
	Proteobacteria	*Enterobacteriaceae*	*Citrobacter*	*Citrobacter sedlakii* NBRC 105722	64	4
	Proteobacteria	*Alcaligenaceae*	*Alcaligenes*	*Alcaligenes* sp. Strain HPC1271	N/A	N/A
	Proteobacteria	*Aeromonadales*	*Aeromonas*	*Aeromonas taiwanensis* LMG 24683	129	14
	Proteobacteria	*Caulobacteraceae*	*Brevundimonas*	*Brevundimonas diminuta* 48290_B02	633	59
	Proteobacteria	*Enterobacterales*	*Serratia*	*Serratia marcescens* subsp. *marcescens Db11*	145	26
	Proteobacteria	*Pseudomonadaceae*	*Pseudomonas*	*Pseudomonas aeruginosa* PAO1	471	90
	Actinobacteria	*Nocardiaceae*	*Rhodococcus*	*Rhodococcus ruber* ASM274172v1	938 *	270 *
Eukaryota	Fungi	*Gloeophllaceae*	*Gloeophyllum*	*Gloeophyllum trabeum*	234	21
	Fungi	*Trichocomaceae*	*Talaromyces*	*Talaromyces islandicus*	49	29

* indicates that the number of enzymes was the summary of enzymes within the species instead of the target strain. N/A indicates that no available information for enzymes was for the target species.

**Table 3:** Ranking of the depolymerization potential of PS-degrading enzymes.

Selected Enzymes		Enzyme Class	Reaction Likely Catalyzed	Potential Carbon Targeted by Enzyme	Enzyme Ranking *
Enzyme Family	Representative Subclass				
Cytochrome P450		Oxidoreductases	Catalyzing the introduction of one atom of molecular oxygen into nonactivated C-H bonds.	β-carbon	High
Monooxygenase	Alkane monooxygenase, 4-hydroxybenzoate 3-monooxygenase, 3-hydroxybenzoate 6-monooxygenase	Oxidoreductases	Incorporating one atom of the oxygen molecule into substrates.	β-carbon	High
Aromatic ring hydroxylase	2-octaprenyl-6-methoxyphenol hydroxylase, 2-octaprenyl-3-methyl-6-methoxy-1,4-benzoquinol hydroxylase, aromatic ring hydroxylating dioxygenase	Oxidoreductases	Incorporating two atoms of dioxygen into the aromatic ring with the dihydroxylation reaction.	U-ring-carbon	Moderate
Esterase		Hydroxylase	Splitting esters into an acid and an alcohol.	Esters	Low
Alpha/beta hydrolase		Hydroxylase	Diverse catalytic functions including hydrolysis, proteolysis, removal of a halogen atom, etc.	Ester and peptide bonds.	Low

* A ranking scale includes high, moderate, and low.

## Data Availability

Data sharing is not applicable to this article.

## Supplementary Materials

The following are available online at https://www.mdpi.com/1996-1944/14/3/503/s1, Figure S1: Phylogenetic tree of DNA sequences of all subclass enzymes from target microorganisms listed in Table 2 including cytochrome P450, monooxygenase, aromatic ring hydroxylase, esterase, and alpha/beta hydrolase, Table S1: Potential PS degrading microorganisms and representative enzymes listed in literatures, Table S2: DNA sequences of higher potential subclass enzymes from target microorganisms including cytochrome P450, monooxygenase, aromatic ring hydroxylase.
