# Supplementary material for: Potential for and Distribution of Enzymatic Biodegradation of Polystyrene by Environmental Microorganisms
Source: Materials (Basel). 2021 Jan 21;14(3):503. doi: 10.3390/ma14030503 (PMC7864516; doi:10.3390/ma14030503)
Supplement: Supplementary file 1 [file materials-14-00503-s001.pdf]

materials

MDPI

Potential for and Distribution of Enzymatic Biodegradation of Polystyrene by Environmental Microorganisms

Liyuan Hou and Erica L.-W. Majumder

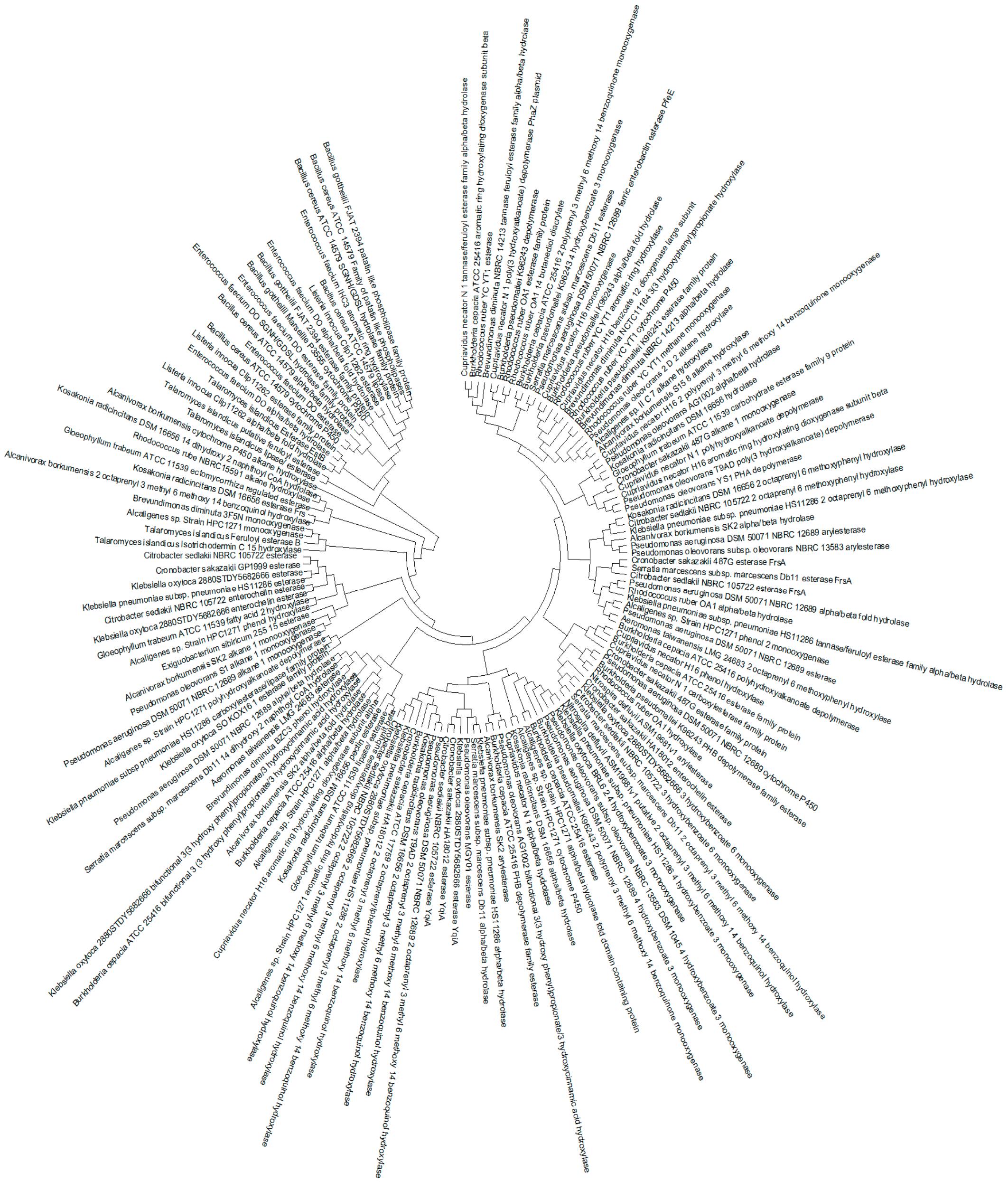

Figure S1. Phylogenetic tree of DNA sequences of all subclass enzymes from target microorganisms listed in Table 2 including cytochrome P450, monooxygenase, aromatic ring hydroxylase, esterase, and alpha/beta hydrolase.

Table S1. Potential PS degrading microorganisms and representative enzymes listed in the literature.

Kingdom	Species	Representative Potential Enzymes
Bacteria	*Bacillus cereus* ATCC 14579	Alpha/beta hydrolase, SGNH/GDSL hydrolase family protein, cytochrome P450
	Cytobacillus gottheilii ASM163634v1	Esterase family protein, cytochrome P450
	Enterococcus faecium DO	Alpha/beta hydrolase, SGNH/GDSL hydrolase family protein, aromatic ring hydroxylase, esterase family protein
	Listeria innocua Clip11262	Esterase family protein, alpha/beta fold hydrolase
	Exiguobacterium sibiricum 255-15	Esterase
	Cupriavidus necator N-1	Alpha/beta hydrolase, carboxylesterase family protein, polyhydroxyalkanoate depolymerase, phenol hydroxylase, monooxygenase, aromatic ring-hydroxylating dioxygenase subunit alpha
	Burkholderia cepacia ATCC 25416	Esterase family protein, alpha/beta fold hydrolase, polyhydroxyalkanoate depolymerase, PHB depolymerase family esterase, 2-polyprenyl-3-methyl-6-methoxy-1,4-benzoquinone monooxygenase
	Burkholderia pseudomallei K96243	PHB depolymerase family esterase, depolymerase, alpha/beta hydrolase, 2-polyprenyl-3-methyl-6-methoxy-1,4-benzoquinone monooxygenase
	Klebsiella pneumoniae subsp. *pneumoniae* HS11286	Alpha/beta hydrolase, carboxylesterase/lipase family protein, 2-octaprenyl-3-methyl-6-methoxy-1,4-benzoquinol hydroxylase, 4-hydroxybenzoate 3-monooxygenase, 2-octaprenyl-6-methoxyphenyl hydroxylase
	Kosakonia radicincitans DSM 16656	Hydrolase, alpha/beta hydrolase, 2-octaprenyl-6-methoxyphenyl hydroxylase, 2-octaprenyl-3-methyl-6-methoxy-1,4-benzoquinol hydroxylase
	Cronobacter sakazakii ASM98282v1	Esterase, esterase family protein, 2-octaprenyl-3-methyl-6-methoxy-1,4-benzoquinol hydroxylase, alkane 1-monooxygenase
	Klebsiella oxytoca ASM102219v1	Esterase, esterase family protein, 2-octaprenyl-3-methyl-6-methoxy-1,4-benzoquinol hydroxylase, bifunctional 3-(3-hydroxy-phenyl)propionate/3-hydroxycinnamic acid hydroxylase, 4-hydroxybenzoate 3-monooxygenase
	Citrobacter sedlakii NBRC 105722	Esterase, 2-octaprenyl-6-methoxyphenyl hydroxylase, 2-octaprenyl-3-methyl-6-methoxy-1,4-benzoquinol hydroxylase, 3-hydroxybenzoate 6-monooxygenase
	Alcaligenes sp. Strain HPC1271	polyhydroxyalkanoate depolymerase, alpha/beta hydrolase, aromatic-ring-hydroxylating dioxygenase subunit beta, monooxygenase, phenol 2-monooxygenase, phenol hydroxylase, cytochrome P450
	Aeromonas taiwanensis LMG 24683	Esterase, 2-octaprenyl-6-methoxyphenyl hydroxylase
	Brevundimonas diminuta 48290_B02	Alpha/beta hydrolase, monooxygenase, phenol hydroxylase
	Serratia marcescens subsp. marcescens Db11	Esterase, alpha/beta hydrolase, 2-octaprenyl-3-methyl-6-methoxy-1,4-benzoquinol hydroxylase
	Pseudomonas aeruginosa PAO1	Esterase, alpha/beta hydrolase, 4-hydroxybenzoate 3-monooxygenase, cytochrome P450, alkane 1-monooxygenase
	Rhodococcus ruber ASM274172v1	Esterase, alpha/beta hydrolase, aromatic ring hydroxylase, cytochrome P450, alkane hydroxylase
Eukaryota	*Gloeophyllum trabeum*	Lipase/ esterase, fatty acid-2 hydroxylase
	Talaromyces islandicus	Feruloyl esterase B, putative feruloyl esterase A

Table S2. DNA sequences of higher potential subclass enzymes from target microorganisms including cytochrome P450, monooxygenase, and aromatic ring hydroxylase.

Enzymes from Species	Accession NO.	
Bacillus cereus ATCC 14579_cytochrome P450	WP_000062123.1	ATGTCAATGAAAAACAAAGTTGGGTTAAGCATAGAGGATGGCATTAATTTAG<br>CTTCAGCTCAGTTTAAAGAAGATGCGTATGAAATTTATAAAGAATCGCGAAA<br>AAAACAACCTATATTATTTGTAAACCAAGTTGAAATCGGTAAAGAATGGCTC<br>ATTACTAGATATGAAGATGCTCTGCCACTTTTAAAAGATAATCGTTTAAAAAA<br>AGATTGGACAAATGTGTTTTCTCAAGATATAAAGAACATGTATCTTTCCGTTG<br>ATAATAGTGACCACTTAACAACACATATGCTAAATTCAGATCCACCTAACCA<br>CAGTCGTTTACGATCTTTAGTTCAAAAAGCTTTTACACCGAAGATGATTGCAC<br>AATTAGACGGAAGAATTCAGAGAATAGCAGATGATTTGATAAGTGATATAGA<br>GCGAAAAGGTACATTAAATCTTGTGGATGATTATTCATTTCCATTACCAATTA<br>TTGTAATAAGCGAGATGCTCGGTATTCCAAAAGAAGATCAAGCGAAATTTAG<br>GATTTGGTCTCATGCTGTTATTGCATCACCAGAAACACCTGAAGAAATAAAA<br>GAGACTGAAAAACAACTATCTGAATTTATTACATATCTTCAATATTTAGTTGA<br>TATTAAAAGAAAAGAGCCAAAAGAAGACTTGGTGAGTGCTTTAATACTTGCA<br>GAGAGTGAAGGGCATAAACTTAGCGCTCGGGAACTATATTCAATGATAATGC<br>TATTAATTGTCGCAGGACATGAGACGACAGTGAATTTAATTACAAATACGGT<br>ATTAGCACTTCTTGAAAATCCAAATCAATTACAGTTATTAAAAGATAATCCAA<br>AACTAATTGATTCGGCTATTGAGGAAGGATTGCGTTATTATTCTCCAGTTGAG<br>GTTACAACTGCAAGATGGGCAGCGGAACCTTTTCAAATTCACCATCAAACAA<br>TACAGAAAGGAGATATGGTTATTATTGCATTGGCTTCAGCGAACCGTGATGA<br>AACAGTATTTGAAAATCCAGAAATATTTGATATTACACGGGAGAACAACCGT<br>CACATTGCCTTTGGTCATGGTAGTCATTTCTGCTTAGGAGCTCCACTTGCGAGG<br>TTAGAAGCAAAGATTGCTATTACTACTTTGTTTAATCGAATGCCTGAACTACA<br>AATAAAAGGGAATCGTGAAGAAATTAAATGGCAAGGTAACTATTTAATGCGT<br>TCTTTAGAGGAATTACCTTTAACTTTCTAG
Bacillus gottheilii Marseille-P3555_cytochrome P450	WP_080848536.1	ATGACTGAAGTAAAACACTTTCCAAAAGAAGAGGGTCTCGATCATAGCCTTG<br>ATTTATTAAAAGAAGGCTATTTGTTTATTACAAATCGCAGTACTGCATTTCAA<br>TCAGATATTTTTGAAACACGCCTGCTGGGAGAGCGGGTTATTTGTCTGCGAGG<br>CAAGGAAGCTGCATCAATCTTTTACGATACGCAGAAATTCAAACGAGAAGGT<br>GCAGCCCCAAATCGAATTCAGAAAACCCTGTTAGGGAAAAACGGGGTACAA<br>TCACTGGATGGTGAAGCACATCAGCATCGGAAGGCAATGTTTATGTCGTTAAT<br>GACACCTGCTTCTCTCCAAAGAATGCGTGCTCTTATAAAAAAAGAGTGGGAC<br>ATTGCGGCCAAGAAGTTTTCACAAGAAAAAGAAATTATCCTTTACGAAGAAG<br>CCAAAAGGGTGCTTTGTAAAGCAGCTTGTGACTGGGCTGGTGTTCCTCTTAAA<br>GAGAACAATGTTGAGGAAACGGCCAATTTACTTGGATTACTATTTGAAACAC<br>CCGCAGCACTCGGCCCTAAGCATTGGCAAGGCAGACACGCACGGACAAAAT<br>TGGAAAAATGGCTTAAGGAATTAGTCATAGAAGTTCGAAACGGCAAATTCCT<br>CCCTCCTGAAGATAAATCCTTATTCATTATTTCAAATCACCGCAATTTGGATG<br>GTGAGCTGCTTGATGCAGACATCGCCGCTGTTGAACTATTAAATATTTTGCGT<br>CCTATTGTCGCGGTAGCCGTGTATATTTGTTTTACGGCGCTTGCTGTATACCAG<br>CATCCAAAAGAAGCAGGCAAACTGCGCGGGTATGATGAAAATCTTCTTCAGA<br>ACTTTGTACAAGAGGTCAGGAGATTTTATCCATTTTTTCCGTTTGCACCAGCA<br>AGAGTAAAAGCGGACTTCACATGGAATGGATATTCTTTTGAGGAAAATACAT<br>TAACACTCCTCGATTTATATGGAACAAACCACCACCCTAAATTATGGGATAA

Enterococcus faecium IHC3_aromatic ring hydroxylase	RCT70246.1	CCCTGAGCTCTTTCAGCCGGATCGTTTTTCAAACTGGAAGGACAGTCCATTCA<br>GTTTTATTCCTCAAGGCGGAGGTGACCATGATTTTGGTCATCGCTGTGCAGGC<br>GAATGGGTGACGATTGAAATTATGAAGGAAACGCTGAATTTTCTTGTGAATA<br>AAATTAGCTTCGCCATTCCAGATCAGGACTTAAGCTATAGCTTTAATGATATT<br>CCAGCTCTTCCTCACAGTAAAATCATTATGAAAGAAATTCATTTAAAATAA<br>ATGATAAATAAAAATCGTGTTAAGGAGTGTCGTACGATGAGCGAAGCAAAC<br>CAAGAACGGTCTGGACAAGAAATAGAACTAATCAAAGAACGTATTCTTGCTG<br>CCTTGGAAATGGTGATTGATCCTGAATTAGGAATCGACATCGTCAATTTAGGC<br>TTGATTTATGATATAGAATTCAATCCTGAAAATGGAGAAACAGTCATTAAGA<br>TGACTCTAACAACTATGGGCTGTCCATTAGCAGATATTTTAACAGAATCTATC<br>CATGACGCATTAAAAGAAGTGCCAGAAGTAACGAAAGAAGAAGTAAAACTA<br>GTCTGGTAG
Cupriavidus necator H16_phenol hydroxylase	WP_010810421.1	ATGAACACCCCGTCGGTTCCCCTGTTCGAAGCCACGCCGCGCTATGTGCGGGT<br>CGAGGGCCGCACCCCCGAAGGGTTTGTGCAGTTCGCCTTCAGCGTGGCCGAC<br>CCGGAGCTCAACGTCGAACTGATCATGCCGGAACCGATGTTCGAAGCCTTCT<br>GCTGCGTCAACCGCGTGCGCTTCCTGCCCCCGCTGGAAACCGCGCCGCAGCC<br>GCAAGCCGACGACTGA
Cupriavidus necator H16_monooxygenase	WP_010810419	ATGTCAGCCACCGCTGCCAACGTCTATATCGCCCTGCAGAACAACGACGACA<br>CCCGACCCATCATCGACGCCATCACCGAGGCCAACCCGCATGCGGTGGTGTC<br>GCAGTTTCCCGCCATGGTCAAGATCGACGCCCCCGGCCACCTGACCATCGTG<br>CGCGAACTGGTGGCCGGCAAGCTCGGCCGCGACTGGGACCTGCAGGAGATC<br>CACCTGAACCTGATCTCGCTGTGCGGAAACATCGACGAAGACGAAGACGCCT<br>TCACGCTGCGCTGGAACGCCTGA
Cupriavidus necator H16_2-polyprenyl-3-methyl-6-methoxy-1,4-benzoquinone monooxygenase	WP_010814779.1	ATGGACACCCTGATCAAAGAATTCGACGTGGCATTGCGCGCCATTGCCGGCG<br>CCACGCGCACCGCGCGCGCCAACCCGGCTGACCGGCTGGCGCCGGACACCG<br>AACAGATGAGCGCCGATGAACGCCGCCACGTGGCCGGGCTGATGCGCATCA<br>ACCATGTCGGCGAGGTCTGCGCGCAGGCCCTGTACCAGGCCCAGAAGCTCAC<br>CGCGCGCGACGGCGCGGTGCGCGCGCAGATGGATGCCGCCGCGCGCGAAGA<br>GGAAGACCACCTGGCCTGGTGCGCCGAGCGCCTGCGCGAGCTGGGCTCGCGC<br>CCGAGCCTGCTCAACCCGCTGTGGTACGCCGGCGCCTTCGCCATCGGCTGGAT<br>GGCCGGCCGCGCCGGCGACCGCGTCAGCCTGGGCTTCGTCGCCGAGACCGAG<br>CGCCAGGTCGAGCACCACCTGGGCGGGCACCTGGACCGCCTGCCCGAGGCC<br>GACGGCCGCTCGCGCGCCATCCTCGAGCAGATGCGCGACGACGAGATCCGCC<br>ACGGCAACGCCGCGCGCGATGCCGGCGGGATCCCGTTACCTGCCCCGGTGCG<br>GGCGCTGATGCGCGGCGCCTCGCGCGTGATGACCACCGCCGCCTACCGGATC<br>TGA
Cupriavidus necator H16_benzoate 1,2-dioxygenase large subunit	WP_013956903.1	ATGATCCCCATCTATCCGGAGGGCAAGGCCCCCGTTCGCCCCGGCGCAAAGC<br>GCCTCGACCTGGATACCTTCCTGGTGGAAGACAAGGCAAGCGGCGACTACCG<br>GCTGCACCGCGCGGCCTTCACTGACGAAGAGCTGTTCGAACTCGAGATGAAG<br>CACATCTTCGAGGGCAACTGGATCTACCTGGCGCACGAAAGCCAGATCCCGA<br>ACAACAACGACTACTACACCACCCATATCGGCCGCCAGCCGGTGGTGATCGC<br>GCGCAATCGCCAGGGCGAACTGAACGCCTTCATCAACGCGTGCAGCCACCGC<br>GGCGCCATGCTGTGCCGCCACAAGCGCGGCAACAAAGCCACCTACACCTGCC<br>CGTTCCACGGCTGGACCTTCAACAACAGCGGCAAGCTGCTGAAGGTGAAGGA<br>TCCCGAGAACGCCGGCTACCCTGACTGCTTCAACAAGGAAGGCTCGCACGAC<br>CTGAAGAAGGTGGCGCGCTTCGAGAACTACCGCGGCTTCCTGTTCGGCAGCC<br>TGAACGCTGACGTGCCGCCGCTGAAGGATTTCCTGGGCGAAGCGGCGCGCGT<br>CATCGACATGATCGTCGACCAGTCGGCCGACGGGCTCGAGGTGCTGCGCGGC

		TCGTCCACCTACACGTTCGAGGGCAACTGGAAGCTGCAGGCCGAGAACGGC<br>GCCGATGGCTACCACGTGTCGGCGGTGCACTGGAACTACGCCGCAACCACCA<br>GCCAGCGCAAGCAGAAGAACGCGCAGGAAGACAAGATCCGCGCCATGGAC<br>GCCGGCAAGTGGGGCCAGCAGGGCGGCGGCTTCTACGCCTTCGAGCATGGCC<br>ACATGCTGCTGTGGACGCGCTGGGCCAACCCGGAGGACCGCCCCAACTTCAG<br>CCGCCGCGAGGAATTCGCCGAGCGTTGCGGCGCCGAGACCGCGGACTGGAT<br>GATCCAGAACTCTCGCAACCTGTGCCTTTATCCCAACGTCTACCTGATGGACC<br>AGTTCGGCTCGCAGATCCGCCTGCTGCGTCCGCTGGCCGTCGACAAGACCGA<br>GGTCACCATCTACTGCATCGCGCCGAAGGGAGAATCCGACGAGGCGCGCGC<br>GCGCCGCATCCGCCAGTACGAGGACTTCTTCAACGTCAGCGGCATGGCCACC<br>CCCGACGACCTCGAGGAGTTCCGCGCTTGCCAGCAGGGCTACGCCGGGCAGG<br>CACTGGCCTGGAACGACATGTGCCGCGGCGCGAAGCACTGGATCGACGGTGC<br>CGACGAGGCCGCGCAGCGCATCGGACTCAAGCCCGTGATGAGCGGGGTGCG<br>CACCGAGGACGAGGGCCTCTACACGGTGCAGCACCGCTACTGGCTGGACGTG<br>ATGAAGAAGGCCGTGGCAGAGCAATCCGCCGAAGCCGCGAATGAAGCCACC<br>GACACCACCGACGCCGCCGCAGGGAGCGCAGCATGA
Cupriavidus necator H16_aromatic ring-hydroxylating dioxygenase subunit alpha	WP_010811430.1	ATGACCGAGACCCCACGACACGAAGCAGGTCAGGCCGCCGATGCGGCCGGG<br>CCCGAACTGGGCCAGTGGCACCCCGTGCTGGCCATCGAACAGGCCGATGGCG<br>ACGGCCCCTTCGCCGCACGGCTCTTTGGCCGCGCGCTGGTGCTGTGGCGCAAT<br>GCCGACGGCTGGCACGCCTGGGACGACCGCTGTCCGCACCGCGGCGCCGCTT<br>TCACGCTGGGCGCCGTGCACGATGGCCTGCTGCATTGCGGCTATCACGGCTG<br>GCGCTTTGCCTCGGGCGGCCGCTGCACGCGCTATCCCGCCCACCCGGAACTG<br>GTGCCTGCGCCGCGCGCCTGCGCCACCACGCACGCGGTGCGCGAGGGCTACG<br>GGCTGTTATGGGTACGCCTTGGCGGCGACGGCACCGAGGCCGTGCCCGAACT<br>GCCGTCCTTCCCCGAGTACCACGACGCCGAATGCCGCCGCGTGGTCTGCGGC<br>CCCTACGACGTTGCCACCTCGGCGCCACGGCTGGTGGAGAACTTCCTCGACA<br>TGGCGCACTTCGGCTATGTCCATGACGGCTACCTGGGTGACCCGGCGCACAC<br>CGAAGTGCCGCCCTATGAAGTGGAAGCCATCGGCGATGCGCGCAAGCCCGA<br>CGCGCTGCGCACCATCGGCTGCCGCGCCTGGCAGCCGCGCGCGCACGCCAGC<br>GCCGCCGGCGGAGCCATGGTCGAGTATGAATACCGCGTGGTTGCGCCCTACG<br>CCGCGATCCTGACCAAGGTGCCCGACCTTGGCGACAACCATCGCGGCGCCAT<br>CGCGCTGTTTATCGGCCCGCGGGACGAGGAATCGAGCCGCGTGTGGTTCGTG<br>ATGTCACTGGTGACAGACGATGACGACGCCAGCCTGCGCGAATTCCAGGACA<br>CCATCTTCCTGCAGGACAAGCCGATCGTGGAATCGCAGCAGCCGCGCCGCCT<br>GCCGTTGCGCACCGGCGTGGAGGTGCCCCAGCCGGCGGACAAGCTGGCAGG<br>CGCGTACCGCCGCTACCTGGCGGCGCATGGGGTGAGGTTCGGCACGCTGGGC<br>TAG
Cupriavidus necator H16_aromatic -ring-hydroxylating dioxygenase subunit beta	WP_041688019.1	ATGAAACCGATGAACTATTCCCTCTATCGCGAGAGCGCCCTCGGCACCGCGC<br>GCGCCATTGAACTGCGACTGGAGATCGAGGCCTTCCACGCGGAATACTGCGC<br>GGTGCTCGACGCCGGCCTGGTGGAGCAATGGCCCCGCTTCTTCACCGAGGAT<br>GCGCTCTACCGGATCACCGCGCGCGAGAATGCCGAGCGCAACCTGCCGGTGG<br>GGCTGGTCTACGCCGAAGGGGTGGGCATGATGCGCGACCGCGCCGCTGCGAT<br>TGCCAATACCCAGATGTTCGCGCCGCGCTACAACCTGCACCTGGTGACCAAC<br>ACGCGGGTCAGCGACGAAACCGCGGACGGCGATATCGTGGCACAGGCCAAC<br>TACATGCTGCTGCAGACACTGGTGGAGGGCCCCACCACGATCCACCTGGCGG<br>GGACCTACTACGACCGCTTCCGCCGCCAGGGTGGCAGCCTGCTGCTGCGCGA<br>GCGGCAGGTTGTCTACGACACCACCATCATCGCCAACGACCTGGTCTACCCG<br>GTCTAG

Burkholderia cepacia ATCC 25416_2-polyprenyl-3-methyl-6-methoxy-1,4-benzoquinone monooxygenase	WP_027788457.1	ATGGTGTTTGATGAACTGATCAGCGAATTCGATCGCGGCCTGCGGTCGCTGACGGGCATTAGCCGGATGAGCCGGCCGGTGCCCGCGCCGGCCGAGGCGCCCGCAGTCGAACTGACGCCCGCGGAGCGTACGCATGCCGCCGGGCTGATGCGCGTGAACCACGTCGGCGAGGTCTGCGCTCAGGCGCTGTACCAGGCGCAGAAGCTCACCGCGCGCACGGCATCCGCGAAGGCGATGTTCGAGGAAGCCGCGCGCGAGGAGGAGGATCACCTCGCGTGGACCGCGCACCGCCTGAAAGAGCTCGACTCGCGCCCGAGCCTGTTGAACCCGCTCTGGTATGCGGGTTCGCTCGCGATCGGCGTGGCGGCTGGCGCGCTCGGCGACAAGGTCAGTCTCGGCTTCATGGCCGAGACCGAACGGCAGGTCGAGAGCCATCTCGAAGGCCATATGTCCGAGTTGCCGGCGGCCGATACCGCGTCGCGCGCGATCGTCGACCAGATGCGTATCGACGAGGTGAAGCACGGCAAGGCCGCCGCCGACGCAGGCGGGATCGAACTGCCGCTGCCCGCGCGAATGCTGATGCGCGCCGCGTCGAAAGTCATGACGAGCACTGCGTACTATCTGTGA
Burkholderia cepacia ATCC 25416_aromatic-ring-hydroxylating dioxygenase subunit beta	WP_027792273.1	ATGACGATTTCATCCGCATTGGCCGACGCACGGCCCGCGACGCGCGGCACCGGAACGGTTGCGGCGACCTTCGCCGATTTCTACGGGCTCGTGGACCTGAACGCGCGCTACGCGTCCGCGCTGGATCGCGGCGACTGGGACGCGTGGCCGGAATTCTTCGAGGAGGACTGCACGTATCAGCTGTTGCCGCGCGAGAACCATGAACGCGACTTTCCGCTTGCGCTGCTCGCGTTCGAGAGCAAGGGGATGCTGAAGGATCGCGTGTACGGCATCAAGGAGACGCTGTTTTACGACCCGTATTACCAGCGCCACCTGATCGGCACGCCGCTCATCACGTATCTCGACGAATCGGTCATCGAGGCCGAGACGAACTACGCGGTGCTGCGTACCAAGAGCGAGCAGATGAGCGACGTGTACAACACCGGTCGTTATCTCGACCGGATACGCCGCACGCCGCACGGGCTGCGTTTCGAGTCGCGCATCTGCGTGTACGACAGCGAGATGGTGCCGAATTCGATCATCTATCCGATCTGA
Burkholderia cepacia ATCC 25416_bifunctional 3-(3-hydroxy-phenyl)propionate/3-hydroxycinnamic acid hydroxylase	WP_027787836.1	ATGAATACCGAATCGAGCGCGCACGGCGACAGCACCGCCGAACACTGCGACGTCGCGATCGTCGGCTACGGCCCGACCGGCCTCGTCGCAGCATCGATGCTGGGCCGCGCCGGCCATCGCGTGATCGTCCTCGAACGCTGGCCGACGCCCTACGGCCTGCCGCGCCTCACGCACATCGACGGCGAAACCGCGCGCATCGTGCAGGCCAGTGCAGACGTCGATCACGCGCTGCGCAACGCGAAGGCCGTCGACACCTACCACTACTGCGACGCGAACGGCGACCTGCTGCTCGAACTGAACTGGACCGGCCGCGCATGCGGCTATCCCGCGCACATCTCGATCTATCAGCCCGACATCGAGGACGCGATCGATGCGCGCGCCAGCGCCTTCAGGAACGTGACGATCCTGCGCGGCTGGGAAGTCGACGCACTGCGGCAGGACGACGACGGCGTGACGCTGACCGCGCATCCGTCCCGCTACGGCCAGGACGCGCAATGGACCGACAGGCCGCGCGTCATTCGCGCGCGCTACGTGATCGGCGCCGACGGCGCGAACAGCCTCGTGCGCCGCACGCTCGGCATCGAACGCTCCGATTTCGGCTGCAACGAACGCTGGCTCAATCTCGATTCGGAAAACAAGCGCGATCTCGGCGACGGCTGCGCGCGCACGACGATCTACTGCGATCCGGCGCGCGCGTACATGCACATGCCGATCGGCACGAAGCGCACGCGTTTCGAGCTGCGCGTGCTGCCGAACGAATCGACGGCCGAATGGGAAACGGAAGAAGCCGGCTGGAAGTGGCTCAACGACCACTACGGCTACGGCCCGGACGACCTGACGCTGCTGCGGCACGTCGTCTACACGTTCGAGACGCGCATGGCCGAACGCTGGCGCGCCGGCCGCGTGCTGCTCGCGGGAGATGCGGCGCACACGATGATGCCGTACATGGGACAAGGCGCGTGCTCCGGCATGCGCGACGGCATCAACCTCGCGTGGAAGCTCGACCTGGTGCTGACCGGGCGCGCGTCCGCCGACCTGCTCGACACCTATGAAGCCGAGCGCCGCCCGCACGTGACGGCGATCACGCAGATGTCGCTGTTTCTCGGGCAGGTCGTCAACGAGGACGATCCGCGCAAGGTCGCCGAGCGCGACGCGGCGTTCCGCTCCGGCAACATGCCGCCGATGCCGCCGTTCCCGAAGCTCGGCCACGGCATCGTGCACGCGCAAGCCGACGGCACGCTGC

Burkholderia cepacia ATCC 17759_2-octaprenylphenol hydroxylase	POM19962.1	TGCCGACGACGGGTGCACCGGCCCCGCAAGGCCGCGCACGGCGCGGCGCGG<br>CCGAGGGCCGTTTCGACGACGTGGTCGGCCAGGGTTTCCAGCTCGTCACGCG<br>CGAGCACCCTGCCCGCCATCTCGACGACGCGCAGCGCGCATTCCTCGCGCAG<br>CTCGGCTGTCACGTGGCCGTGCTGGCCGACGACGTGTGCGCGCCGGATGCCG<br>TGGTCGATCTCGACGGCGAGCAGCACGCGTTCATGCACGCGCACGGCATCGC<br>CGCGTATATCAAGCGGCCCGATTTCATCGTGTTCGGTTCGGTGACGGACCTGC<br>GCGATCTCGGCGCGCTCGTCGACGCGCTGCGCGCCGCACTGCACTGGTCTGC<br>CGTTTCGAGCGAGCCTGCCGCGGCTTCGGCAGCCGTCACCCACGCATCGTGA<br>ATGACTGCCCACCACTCCTTCGACGTCGCCGTGGTCGGCGGCGGGCTCGTCGG<br>CAAGACGGCCGCGCTCGCACTGACCCAGTCCGGCTACAAGACTGCCTTGCTC<br>GCGCAGCCGGCCGCGCCGCGCCCCGCCGATCTCGTATTCGACACGCGCGTCT<br>ACGCGCTGTCTTCCAGTTCGCAGGCGTTGCTCGAGCGGCTGCGGGTCTGGCAG<br>GCGCTCGACCATGGCCGCCTCGCACCGGTCTACGACATGCGCGTATACGGCG<br>ATGCGCACGCGGAACTGCATTTCTCCGCGTACCAGGCATCCGTGCCGCAGCT<br>CGCGTGGATCGCCGAATCGTCGCTGGTCGAGGCGTCGCTTGACGCCGCGCTG<br>CGGTTCCAGCCGAACCTCACGTGGTTCGAGGCGCGCGCGCAGGGCTTCGACG<br>TACACGACGACGCAGCCGTGCTCACGCTGTCGTCGGGGCAGGTGCTGGAAGC<br>GGATCTCGTCGTCGGCGCGGACGGCGCGCATTCGTGGGTGCGCTCGCAAATG<br>GGGGCCAAAGTCGAACGGCGCGACTACCGGCAAACCGGTGTCGTCGCGAAC<br>TTCAAGGCGTCGCTGCCGCATCGCGAGACGGCCTACCAGTGGTTCCACGAAG<br>GCGAGATCGTCGCGCTGCTGCCGCTGCCGGACGGCCATGTGTCGCTCGTCTGG<br>TCCGCGCACACCGCGCATGCGGATGAATTGCTCGCACTCGATCCGGCGCAGC<br>TCGCGGCCGAGGTCGAGCGCGTGTCTCATGGCCAGGTCGGCACGCTCGACTG<br>CGTGACGCCGGCCGCTGGCTTCCCGCTGGCGCTGCAGACGGTCGACAAGCTG<br>ATCGCGCCGCGCGTCGCGCTGGTCGGCGATGCCGCGCACCTGATCCACCCGC<br>TCGCGGGACAGGGGATGAACCTCGGCCTGCGCGACGTGGCGGCGCTTGCCGA<br>TGCGATCGCCGGCAAGGAAAGCTTCCGCAACCTCGGCGATACGGTGCTGCTG<br>CGTCGCTACGAGCGTTCGCGCCGCGAGGACATCCGCGCGCTGATGGTCGCAA<br>CCGACGGCCTGCAGCGGCTGTTCGCGGTGCCGGGCTCGCTCGCGAAGGCCGT<br>GCGCAATGCGGGCATGGCCTTCGTCGGTGCGCAGCCGCTCGTGAAGCGCTGG<br>CTCGTGTCGGCCGCGCTCGGCTGA
Burkholderia pseudomallei K96243_2-polyprenyl-3-methyl-6-methoxy-1,4-benzoquinone monooxygenase	WP_004527787.1	ATGCGCACGCAAGTCGCCATCATCGGCGCCGGGCCGTCCGGGCTGCTGCTTT<br>CCCACCTGCTCCGTCTGCAAGGCGTCGATTCCGTCGTGCTCGAGACGCGTTCA<br>CGCGAATACTGCGAGAACCGGATCCGCGCCGGCGTGCTGGAGCAGGGCACC<br>GTCGACACGCTGAACGATGCGGGCCTCGGCGCGCGGATGCGGCGCGAAGGG<br>CTCGTGCATTGCGGCATCGAGCTGCTGTTCGACGGCCGCCGCCACCGGATCG<br>ATTTCGGCGAGCTGACGCCCGGCCGCGCGATCACCGTCTACAGCCAGCACGA<br>AGTCGTGCGCGACTTGATCGAGGCCGCGCTCGAGCACGGCCAGCCGATCCAC<br>TTCGACGTGCGCGACGTCGCGCTGCACGACGTCGCGACCGAGCGGCCGTCGG<br>TGACGTTCATGCACGCGGACGGTCGCCCGGATCGGATCGATTGCGACTATGT<br>CGCCGGCTGCGACGGCTTTCACGGCGTCGCGCGCCAGGCGATCCCCGCCGAG<br>CGGCGGCGCACGTTCGAGCGCGTGTACCCGTACGCCTGGCTCGGCATCCTCG<br>CCGATGCGGCGCCGTCGCTCGACGAGCTCGTCTATGCGCATCACGCGCGCGG<br>CTTCGCGCTGTTCTCGATGCGCTCGCCGACCGTCACCCGACTGTACCTGCAAT<br>GCCGGCCCGACGAGAACCTGGCCGAATGGCCGGATGCGCGCATCTGGGAGG<br>AACTGGGCACGCGCTTCGAAAACGACGGCGGCTGGACGCCGACCCCAGGCC<br>GCATCACGCAGAAGAGCGTGACGCCGATGCGCAGCTTCGTGTCGGAGACGAT<br>GCAGCACGGCCGGCTGTTCCTCGCGGGCGACGCCGCGCACATCGTGCCGCCG

		ACCGGCGCGAAGGGGATGAATCTCGCGGTCGCCGATGTCCGGGCGCTGTCGC<br>GCGCGCTCGTCGCGCGCTACCGTCACGGCCGCGGCGATCTGCTCGATGCGTAT<br>TCGTCAACCTGTCTGGAACGGATATGGCGTGCCGAGCATTTTTCGTATTCCAT<br>GACGAACATGCTGCACCCGTGCGCCGACGATTCGCCGTTCGTCAATCGGCTG<br>AAGCTCGCCGAGCTCAGGTACGTGACGCGTTCGCGCGCGGCCGCGCAGTCGC<br>TCGCGGAGAACTACGTCGGCCTGCCGTTCGACGAAACCGCGGGCGATGCGCC<br>GTCGTTTGGCGAGGCGGCCGCCGCATGA
Burkholderia pseudomallei K96243_4-hydroxybenzoate 3-monooxygenase	WP_004556451.1	ATGGTGTTTGACGAGTTGATCACTGAATTTGACCGTGGGCTGCGCTCGATCGC<br>GGGTGTGAGCCGGATGAGCCGCCCGGTGCCCAAGCCCGCAGCCGCGGCGCC<br>TGCCGAGCTTTCCGCAGCCGAGCGCAAGCATGCGGCGGGGTTGATGCGGGTG<br>AATCACGTCGGCGAAGTCTGCGCGCAGGCGCTCTATCAGGCGCAGAAGCTCA<br>CGACGTCTTCGGCCGGGCTGAAGGAGATGTTCGAGCATGCGGCGCGCGAGGA<br>AGAGGACCATCTCGCGTGGACCGCGCATCGATTGAAAGACCTCGATTCGCGG<br>CCGAGCCTGCTGAATCCGCTCTGGTATGCGGGCGCGCTCGCGATCGGCGTCGT<br>CGCCGGGCGTCTCGGCGACAAGATGAGCCTCGGCTTCATGGCCGAGACGGAG<br>CGGCAGGTGGAGAGCCACCTTGACGGCCATCTGTCGGAGCTGCCGGCGGCCG<br>ACGTCGAGTCGCGCGCGATCGTCGAGCAGATGCGCGCCGACGAGGTCAAGC<br>ACGGCAAGTCGGCGACCGACGCCGGCGGCATCGAGCTGCCGATGCCGGCGC<br>GCATGCTGATGCGCGCGGCATCCAAGGTCATGACAAGCACTGCATACTATCT<br>GTGA
Klebsiella pneumoniae subsp. pneumoniae HS11286_2-octaprenyl-3-methyl-6-methoxy-1,4-benzoquinol hydroxylase	WP_002894733.1	ATGACAATTCACGTAACAGATGTCGCCATCGTGGGCGGCGGCATGGTCGGCG<br>GCGCCCTGGCGTTAGGGCTGGCGCAACAGGGATTTACCGTGACGGTGCTTGA<br>GAAAGCGGCGCCACCGGCGTTCGATCCGGCATCCGCCCCGGATGTGCGGATC<br>TCGGCGATCAGCGCGGCTTCGGTCGGCCTGCTGAAGAGTCTCGGCGTCTGGG<br>ATGCCATACGGGCGATGCGCGTCCACGCGTATCGCCGTCTGGAGACCTGGGA<br>GTGGGAGAGCGCGCACGTCGCCTTCGACGCCGCGGAGCTCAAGCTGCCGGA<br>GCTGGGCTATATGGTCGAGAACAAGGTTCTGCAATGGGGCCTGTGGCAAGCG<br>CTGGCGGCGCATGAGGCGGTTACCCTGTGCGTCGGTAGCGAACTGAAAACGA<br>TGCAGCGCGGCGAGACGCAGACGGCGTTGCATTTGCGCGAGGGGGAGACGA<br>TCCACGCCCGGCTGGTTATCGGCGCCGACGGCGCTAACTCCCAGGTGCGGGA<br>GATGGCCGGCATCGGCGTGCACGCCTGGCAATATCAGCAGTCCTGTATGCTG<br>ATCAGCGTCGAATGCGCCGACGATCCCGGCGACAGCACCTGGCAGCAGTTTA<br>CTCCCAGCGGCCCACGCGCCTTCCTGCCGCTGTTCGACCACTGGGCTTCGCTG<br>GTCTGGTACGACGCCCCGGCGCGGATCCGTCAGCTGCAGAGCATGACCATGG<br>CGCAGCTCCAGCAGGAGATCGCCAGTCACTTTCCGGCGCGGCTGGGACGGGT<br>GACGCCGCAGGCGGCCGGCGCCTTCCCGCTCACCCGGCGTCATGCGCTGCAG<br>TATGTTCAGCCCGGCCTGGCGCTGGTGGGCGATGCGGCGCACACCATTCATCC<br>ACTGGCGGGGCAGGGGGTTAACCTTGGCTATCGTGATGTCGATGCCCTGCTG<br>GAGATTCTGGCGGAAGCGCGCGGCCGCGGCGAAGACTGGGCCAGCCTGCCG<br>GTGCTGAAGCGCTACCAGGCGCGGCGGCGAGCGGATAATTTTATCATGCAGT<br>CGGGGATGGATCTGTTTTATGCCGGTTTCAGCAATGACCTGGCGCCGGTGCGG<br>ATGCTGCGTAATATTGGCCTGATGGCGGCTGAACGCGCCGGGGTGCTGAAGC<br>GTCAGGCGCTGAAGTATGCCTTAGGCTTGTAA
Klebsiella pneumoniae subsp. pneumoniae HS11286_4-	WP_004151384.1	ATGAAGACACAGGTCGCGATTATTGGCGCGGGCCCCTCCGGGCTGCTGCTGG<br>GGCAGTTGCTGCACAACGCCGGGATCCATACCGTGATCCTTGAGCGTCAAAC<br>GCCGCAGTATGTGCTGGGGCGCATCCGCGCGGGAATTCTGGAAAGCGGTACC<br>GTTGATCTGCTGCGCGAAGCGGGCGTGGCGCAGCGGATGGATGCCGAAGGCC<br>TGGTGCATCATGGGGTGGAGTTTCTGTTTGACGGCCAGCGGGTGCCCGTCGCA

hydroxybenzoate 3-monooxygenase		CTGAGCGAGCTAACCGACGGTAAGAGCGTAATGGTTTATGGCCAGACCGAA<br>GTCACCCGCGATCTGATGGCTGCCCGGGCCGCCAGCGGCGCGCCGATCGTCT<br>ATGGCGTGAGCGAGGTGGCTATCCATGACGCCAAAAGCGACCGGCCGACGA<br>TCACCTACCTCAGCGAAGGGGAAACCTGCCGTCTGGAGTGCGATTTTATTGCC<br>GGTTGCGACGGTTTCCACGGCGTATCGCGGCAAAGTATTCCAGCTGGCATTTT<br>GCAGACCTACGAGAGCGTGTGGCCGTTTGGCTGGCTAGGCCTGCTGGCGGAT<br>ACGCCGCCGGTCAATCCGGAGCTTATTTATGCGCACCATCAGCGGGGGTTTGT<br>ATTGTGCAGTCAACGCTCACTGACTCGCAGCCGCTATTATCTGCAGGTTCCGC<br>TGAGCGATAAGGTTGAAGCATGGTCGGACGAGCGCTTCTGGCAGGAGCTGAA<br>AAGCCGCTTGCCGGAGGAGCTGGCATCCAGGTTAGTGACTGGTCACGCACTT<br>GAGAAGAGCATTGCCCCCCTGCGTAGCTTCGTCGTGGAGCCGATGCAATATG<br>GGCGGTTGTTCCTGGTGGGGGATGCGGCGCATATCGTTCCGCCAACCGGGGC<br>GAAAGGATTAAATCTGGCGGCATCGGACGTGAACTATCTGTGGCGGATTCTG<br>CGCGAATACTACCATCGTGGCCGCAGCGATCTGCTGGCGGCCTACTCGCAAC<br>TGGCGCTGGATCGGGTCTGGAAAGGGGAACGCTTTAGCTGGTTTATGACCCG<br>CCTGCTGCATGATTTTCCGGATCAGAACGCGTTCGACGCTAAAATGCAGGCC<br>GCCGACCGCCGCTACTATCTGGGCTCGCGCGCCGGGCTGACCACCATCGCGG<br>AAAACTACGTGGGCTTGCCCATGGAGCGTGTCGCCTGA
Klebsiella pneumoniae subsp. pneumoniae HS11286_2-octaprenyl-6-methoxyphenyl hydroxylase	WP_002916482.1	ATGAGCGTGCTGATCGTCGGCGGCGGGATGACCGGGGCGACGCTCGCGCTGG<br>CCATCTCCCGTTTGACCGGCGGCGCGCTGCCGGTCCACCTCATTGAAGCGCAG<br>GATCCGCATTCTTCACGCCACCCTGGCTTTGACGACCGGGCGATCGCCCTGGC<br>TGCGGGCACCTGCCAGCAGCTGGCGCGTATCGGTATCTGGCAACGCCTCGCC<br>GAACGCGCGACGCCCATCCAGCGAGTACACGTCAGCGACCGCGGTCATGCC<br>GGGTTTGTGAATCTGGCGGCCGCGGATTATGGCCTGTCGGCGCTGGGCCAGGT<br>GGTTGAACTCCACGATGTCGGCCAGCGGCTGTTTGGTCTGCTGCGCGAGGCGC<br>CAGGCGTCACGCTGCACTGTCCGGCGAAAGTGGAAGCGGTCAGTCGCAGCCA<br>GGAGAGCGTCAGCCTTACGCTTGAGGGCGGCGAAATCATTAACGGTAAGCTG<br>CTGGTGGCGGCGGACGGTTCCCGTTCGGCACTGGGCGCCCGCTGCGGCATCA<br>GCTGGCAGCAGCAGCCGTATGAGCAGATAGCGATCATTGCTAACGTCAGCAC<br>CGCGTTGCCCCACGAAGGTCGCGCCTTCGAGCGCTTTACCGAGCATGGCCCG<br>CTGGCGATGCTGCCGATGTCGCAAGGGCGCTGCTCGCTGGTGTGGTGTCATCC<br>GCAGTCGCGCCGCGATGAGGTGCAGAGCTGGTCTGACGAACGCTTTTGTCAG<br>GAGCTGCAGCAGGCGTTCGGCTGGCGGCTGGGGCGTATTACCCACGCCGGGA<br>AGCGCAGCGTTTATCCGCTGGCCCTCACCACCGCCAGCCGCGCGGTGTCGCA<br>CCGCCTGGCGCTGGTCGGCAACGCGGCGCAGACGCTGCACCCGATCGCCGGG<br>CAGGGCTTTAATCTCGGCCTGCGCGATGTGATGAGCCTGGCGGAATTACTCGC<br>TGACGCCCATCTCAGCGGCGAAGATGTCGGTCATTATCCACTGCTGTGCCGCT<br>ATCAGGCGCGCAGGGCAGGGGATAAAGCCGCCACCATCGGCGTGACCGATG<br>GGCTGGTGCATCTGTTTGCCAACCGTTGGGCGCCGCTGGTGGCGGGGCGCAA<br>CGTCGGTCTGATGGCGATGGAATTATTTACCCCGGCGCGCGATGCGCTGGCGC<br>AGCGTACCCTCGGTTGGGTTCCCCGTTAA
Kosakonia radicincitans DSM 16656_2-octaprenyl-6-methoxyphenyl hydroxylase	WP_007370239.1	ATGAGTGTGATTATTGTTGGCGGCGGCATGGCCGGTGCGACGCTCGCGCTGGT<br>CATTTCACATCTGACGCAGGGGAAATTACCGGTTCACCTGGTAGAGGCTACA<br>GCACCGGGTGCTCACCATCATCCTGGTTTTGATGCCCGTGCTATTGCGCTGGC<br>GGCAGGCACGTGCCAGCAGCTTGCGCGCGTCGGGATCTGGCAGGCGATAGCC<br>CGTTGTGCAACGCCCATTTCAACCGTTCATGTCAGCGATCGTGGTCACGCCGG<br>TTTTGTCACGCTGGATGCGCAGGATTATCACCTTGATGCGCTGGGCCAGGTGG<br>TTGAGCTGCATGATATCGGCCAGCGGCTCTTTACCTTGCTGCAAAATGCGCCG

		GGCGTGACGCTGCATTGCCCCGATCGGGTCAGCAGTTTTACCCGTAATGAGC<br>AACAGGTATCGGTAACGCTGGAAAGCGGCGAGCTGATTATTGGCCAGTTGCT<br>GGTCGCGGCGGACGGTTCCCGTTCGGGCATGGGCGAGCAACTGGGGATCCTC<br>TGGCAGAAAACGCCCTATCACCAGGCCGCAGTCATTGCCAACGTCACTACCT<br>CTGTTCCGCACCAGGGACGCGCATTCGAACGTTTCACCGAGCACGGGCCGCT<br>GGCGATGTTGCCGATGTCGCAGGGGCGCTGCTCACTGGTGTGGTGTCATCCGC<br>TGGAAAAACAGCAGCAGGTGACTGGCTGGTCTGACGAACGTTTTTGCCATGA<br>GCTACAAAAAGCTTTCGGCTGGCGTCTGGGACGTATTGAACACGCCGGAAAA<br>CGCAGCGTTTATCCCCTGATGTTGACTAACGCGTCGTCGCCGGTTTCTCATCGC<br>GCGGTGCTGGTCGGCAATGCGGCTCAGACGCTGCATCCTATCGCCGGACAAG<br>GGTTTAACCTCGGCATGCGCGATGTTATGACGCTGGCGGAAACGCTGGCGGA<br>GGCATGGGCGAAGCAGAAAGATGTCGGCTGTTATCCGGTGCTGACGCGCTAC<br>CGTCACCAGCGCCAGGCGGACAAAAGCGCCACCATAGGCGTTACCGACGGG<br>CTGGTGCATCTGTTTGCCAACCGCTGGGCGCCGCTTGTCGTCGGGCGCAATAT<br>CGGGCTGATGGCGATGGAACTATTTACCCCGGCGCGCGATGTGCTGGCGCAG<br>CGTACCCTCGGTTGGGTAGCCCGTTAA
Kosakonia radicincitans DSM 16656_2-octaprenyl-3-methyl-6-methoxy-1,4-benzoquinol hydroxylase	WP_007373869.1	ATGACACATCACACTGAAGTCGCCGTCGTTGGCGGTGGCATGGTCGGCGGAG<br>CGCTGGCGCTGGGGCTGGCGCAACATGGATTTACCGTTACGGTAATTGAGCA<br>GCATGCGCCGCCCACGTTTGATGCGACGGCGCAGCCGGATGTGCGTATTTCG<br>GCAATTAGCGCCGCATCGGTGGCGTTGCTGCGCGGGTTGGGTGTCTGGGAAC<br>GTGTGCAGGCGATGCGCTGCCACCCGTATCGCCAGCTGGAAACCTGGGAGTG<br>GGAAAACGCCCATGTGATATTTGACGCCCGCGAGCTGAAATTGCCGCTGCTC<br>GGTTATATGGTTGAAAACAATGTCCTGCAACGTGCGCTGTGGGACGCGCTTG<br>AGGCGCATCCGCAGGTCACGTTACGTGTGCCCGCGGCACTCAGCGGATTACA<br>TACGGATGGTGATCATCACCAACTGACCTTCGAGGAGGGCGAGTCGTTAACG<br>GCGAAGCTGGTGGTTGGCGCGGATGGCGCTAACTCCCGCGTTCGGCAGGCGG<br>CCGGAATTGGCATTCACGCCTGGCAGTATCAGCAATCCTGTATGTTGATTACC<br>GTTGAGTGCGAAGCTGAGGCAGGCGAGAGCACCTGGCAACATTTTACTCCCG<br>ATGGGCCGCACGCCTTTTTACCGCTGTTTGGCAACTGGGCCTCGTTGGTATGG<br>TATGACACGCCTGCGCGTATTCGCCAGTTACAGACGCTGACTATGGCGCAGC<br>TGGAGCAGGAGATCGTTCGCAACTTTCCTCCGCGACTGGGGCGCGTGAAGCC<br>GGTTGCGGCGGCGGCATTCCCGCTGGCGCGCCGTCATGCGTTGCAATATGTTC<br>GCCCGGGCCTTGCGCTGGTGGGCGATGCGGCACATACCATTCATCCGCTGGC<br>CGGGCAGGGCGTCAATCTGGGCTATCGTGATGTTGATGCCTTGCTGGAAGTGC<br>TTACCGGGGCACGTACGCACGGCGAAGCCTGGGCCAGTCACGCGATTTTAAA<br>ACGCTATCAGACGCGTCGAATGGCGGATAACTTTATTATGCAAAGCGGTATG<br>GATCTGTTCTATGCCGGGTTCAGCAACAACCTTGGCCCGGTCAGGTTGTTGCG<br>TAATATTGGTCTGATGGCGGCGGAGCGTGCCGGAGTGCTGAAACGTCAGGCG<br>CTGAAATATGCTTTAGGACTATAG
Cronobacter sakazakii HA18012_2-octaprenyl-3-methyl-6-methoxy-1,4-benzoquinol hydroxylase	WP_166492126.1	ATGACAAATCAACCAATTGAAGTCGTCGTTGTCGGCGGGGGCATGGTGGGTG<br>CCGCCGCCGCGCTTGGGCTTGCGCAAAATGGCTTTCAGGTCGCCGTCGTGGA<br>ACATGCCGCGCCGCCGCCGTTTTCACCGCAAAACCCGCCGGACGTGCGGATC<br>TCGTCGATCAGTTGCGCGTCGGTGGATCTGCTGCGCGGGCTGGGCGTCTGGCA<br>GCAGGTGCTTGAGATGCGCGCGCATCCTTATCGCCGCCTCGAAACCTGGGAG<br>TGGAACGAGGCGCGGGTTGAATTCAGCGCCGATGAACTGCAACTGCCGGAG<br>CTGGGCTATATGGTAGAAAACAGCGTGCTGCAACGCGCGCTGTGGGAAGCGC<br>TGGAGGCGCATCCGGCGGTGCAACTACTGTGCCCGGCGACGCTCACGCAGAT<br>GACGCCGTCGCAAAACGGGTATCAGCTCTCGCTTACTAATGGCGACACATTA

		CAGGCGCGCCTGGTGATCGGTGCGGACGGTGCCAGCTCGCAGGTACGTAAAT<br>GGGCAGGTATCGGCGTTAACGCCTGGCAGTATCAGCAGTCATGCCTGTTGATT<br>AGCGTGGAATGCGAGCAGCCGCCCGGCGACAGCACCTGGCAGCACTTTACCC<br>CGAACGGGCCGCATGCGTTTTTACCGTTGTTTGATAACCGTGCGTCGCTCGTC<br>TGGTATGACCGCCCGGCACGTGTTCGTCAGCTTCAGGCGATGACCATGACAC<br>AGCTTGGGCGAGAGATTGCCGCCGCATTTCCTGCCCGGCTTGGCCGCGTGAC<br>GCCGGTCGCCTGCGGCGCGTTTCCGCTGGTGCGTCGCCATGCGCTGCGTTATG<br>TGCAGCCGGGCGTCGCGCTAATTGGCGACGCCGCGCATACCATTCATCCGTT<br>GGCGGGGCAGGGGGTGAATCTCGGTTATCGCGATGTGGACGCGCTGCTGAAG<br>GTGGTGATTGATGCCCGCAATGCGGCGGAAGACTGGGCTTCCGGCGCGGTGC<br>TTAAGCGCTATCAGCGTCGTCGTATGCCGGATAACCTGCTGATGCAAAGCGG<br>TATGGATCTTTTCCATGCCGGGTTTACTGCGAAACTGAAACCGCTGCGCGTGC<br>TGCGCAATCTTGGGCTTATCGCCGCCCAGCGCTCAGGCGTGCTGAAACGCCA<br>GGCGCTGCGGTATGCGCTCGGTTTATGA
Cronobacter sakazakii 487G_alkane 1-monooxygenase	WP_105944427.1	ATGCGCTGCCCGCAGGCGTCGCGCCGCGCATTATGGCCTCGCGCACCGCGTTT<br>TGTGACGGATAACAGTGACGACGCCCGCCGCTTCGCGCAAACCGGGCTGGCG<br>GCTCAGGCCGCGCAGTTCCGCGCGCAGGGCCACCGGCTTACGGGCGAGACG<br>CTGGAAGATTATATCGCCGCGTTCGATGTGCATCTCGGCACCCCGCGGGAAA<br>CGCTCGCCACGCTGTCGCGCGATACGGCGCTCGCACGCGCCACCGATCTGTC<br>GTTCCAGGTGCATTCCATCGATCCGCCGCACCCGTTTATCTTGCGCTCCATCTC<br>ACTGCTGGCGCAGCAGGTGGCCCCCGCGCTTGGCTGGAAACAGGCCGCACCG<br>GGCGCTGATGCCTCCACCCTTAACCCGATCAAGGAAACCTTATGA
Klebsiella oxytoca 2880STDY5682666_2-octaprenyl-3-methyl-6-methoxy-1,4-benzoquinol hydroxylase	WP_022645356.1	ATGACAAATCAACCAACGGAAATTGCCATTGTCGGCGGAGGAATGGTCGGC<br>GGCGCACTGGCGCTGGGGCTGGCACAGCACGGATTTGCGGTAACGGTGATCG<br>AGCACGCAGAACCTGCACCGTTTGTCGCTGACAGCCAGCCTGACGTGCGGAT<br>CTCGGCGATTAGTGCGGCTTCGGTATCATTGCTTAAAGGTTTAGGCATCTGGG<br>ACGCGGTACAGGCTATGCGTTGCCATCCTTACCGCAGACTGGAAACGTGGGA<br>GTGGGAAACGGCGCATGTGGTGTTTGACGCCGCTGAACTTAAGCTACCGTTG<br>CTTGGCTACATGGTGGAAAACACTGTCCTGCAACAGGCGCTGTGGCAGGCGC<br>TGGAAGCGCATCCGAAAGTAACGTTACGTGTGCCAGGCTCGCTGATTGCATT<br>ACATCGAGATAATGATCTTCAGGAGCTGGAGCTGAAAGGCGGTGAAGTGATC<br>CAGGCGAAGCTGGTGATTGGCGCTGACGGTGCAAATTCGCAGGTGCGGCAAA<br>TGGCGGGAATTGGCGTTCATGCCTGGCAGTATGCGCAGTCGTGTATGTTGATT<br>AGCGTACAGTGCGAGAATGATCCCGGCGACAGCACCTGGCAGCAGTTTACTC<br>CGGACGGGCCGCGTGCGTTTTTGCCGCTGTTTGATAACTGGGCGTCGCTGGTG<br>TGGTATGACTCTCCGGCGCGTATTCGCCAGTTGCAGAATATGAATATGGCGCA<br>GCTCCAGGCGGAAATCGCGAAGCATTTCCCGTCGCGTCTGGGTTACGTGACA<br>CCGCTTGCCGCTGGTGCGTTTCCGCTGACACGTCGCCATGCGTTGCAGTATGT<br>GCAACCAGGGCTTACGCTGGTGGGCGATGCCGCGCACACCATTCATCCGTTG<br>GCGGGGCAGGGGGTTAACCTCGGTTATCGCGATGTTGATGCGCTGATTGACGT<br>TCTGGTGAATGCCCGCAGCTACGGTGAAGCGTGGGCCAGTTATCCTGTCCTCA<br>AGCGTTACCAGATGCGGCGTATGGCGGATAACTTCATTATGCAAAGCGGTAT<br>GGATTTGTTTTATGCCGGATTCAGCAATAATCTGCCACCACTGCGTTTTATGCG<br>TAATCTCGGATTAATGGCGGCGGAGCGTGCTGGCGTGTTGAAACGTCAGGCG<br>CTGAAATATGCGTTAGGGTTGTAG
Klebsiella oxytoca 2880STDY568	WP_001577817.1	ATGGCAATACAACACCCTGACATCCAGCCTGCTGTTAACCATAGCGTTCAGG<br>TGGCGATCGCTGGTGCCGGTCCGGTCGGGCTGATGATGGCGAACTATCTCGG<br>CCAGATGGGCATTGACGTGCTGGTGGTGGAGAAACTCGATAAGTTGATCGAC

2666_bifunctional 3-(3-hydroxy-phenyl)propionate/3-hydroxycinnamic acid hydroxylase		TACCCGCGTGCGATTGGTATTGATGACGAGGCGCTGCGCACCATGCAGTCGG<br>TTGGCCTGGTCGATAATGTTCTGCCGCACACTACGCCGTGGCACGCGATGCGT<br>TTTCTCACCCCAAAAGGTCGCTGTTTTGCTGATATTCAGCCAATGACCGATGA<br>ATTTGGCTGGCCGCGCCGTAACGCCTTTATTCAGCCTCAGGTCGATGCGGTAA<br>TGCTGGAAGGATTGTCGCGTTTTCCGAATGTGCGCTGCCTGTTTTCCCGCGAG<br>CTGGAGGCCTTCAGCCAGCAAGATGACGAAGTGACCGTGCACCTGAAAACG<br>GCAGAAGGGCAGCGGGAAACGGTCAAAGCCCAGTGGCTGGTGGCCTGTGAT<br>GGTGGGGCAAGTTTTGTCCGTCGCACCCTGAATGTGCCGTTTGAAGGTAAAAC<br>TGCGCCAAATCAGTGGATTGTGGTAGATATCGCCAACGATCCGTTAAGTACG<br>CCGCATATCTATTTGTGTTGTGATCCGGTGCGCCCGTATGTTTCTGCCGCGCTG<br>CCTCATGCGGTACGTCGCTTTGAATTTATGGTGATGCCGGGAGAAACCGAAG<br>AACAGCTGCGTGAGCCGCAAAATATGCGCAAGCTGTTAAGCAAAGTGCTGCC<br>TAATCCGGACAATGTTGAATTGATTCGCCAGCGTGTCTACACCCACAACGCG<br>CGACTGGCGCAACGTTTTCGTATTGATCGCGTACTGCTGGCGGGCGATGCCGC<br>GCACATCATGCCGGTGTGGCAGGGGCAGGGCTATAACAGTGGTATGCGCGAC<br>GCCTTTAACCTCGCATGGAAACTGGCGTTGGTTATCCAGGGGAAAGCCCGCG<br>ATGCGCTGCTCGATACCTATCAACAAGAACGTCGCGATCACGCCAAAGCGAT<br>GATTGACCTGTCCGTGACGGCGGGCAACGTGCTGGCTCCGCCGAAACGCTGG<br>CAGGGTACGTTACGTGACGGCGTTTCCTGGCTGCTGAATTATCTGCCGCCAGT<br>AAAACGCTACTTCCTCGAAATGCGCTTCAAGCCGATGCCGCAATATTACGGC<br>GGTGCGCTGGTGCGTGAGGGCGAAGCGAAGCACTCTCCGGTCGGCAAGATGT<br>TTATTCAGCCGAAAGTCACGCTGGAAAACGGCGACGTGACGCTGCTCGATAA<br>CGCGATCGGCGCGAACTTCGCGGTAATTGGCTGGGGATGCAATCCACTGTGG<br>GGGATGAGCGACGAGCAAATCCAGCAGTGGCGCGCGTTGGGCACACGCTTC<br>ATTCAGGTGGTGCCGGAAGTGCAAATTCATACCGCACAGGATAACCACGACG<br>GCGTACTACGCGTGGGGGATACGCAAGGTCGCCTGCGTAGCTGGTTCGCGCA<br>ACACAATGCTTCGCTGGTGGTGATGCGCCCGGATCGCTTTGTTGCCGCCACCG<br>CCATTCCGCAAACCCTGGGCAAGACCCTGAATAAACTGGCGTCGGTGATGAC<br>GCTGACCCGCCCTGATGCCGACGTTTCTGTCGAAAAGGTAGCCTGA
Klebsiella oxytoca 2880STDY5682666_3-hydroxybenzoate 6-monooxygenase	WP_032728552.1	ATGGCAAAAGTGATGCGAGCAATCATTGTCGGCGGCGGTATTGGCGGGGCAG<br>CCACGGCGCTTTCGCTGGCGCGCCAGGGTATCAAGGTGATGCTGCTGGAAAA<br>GGCGCATGAAATCGGCGAAATTGGCGCCGGGATCCAGCTTGGCCCAAACGC<br>GTTCTCGGCGCTGGACAGCCTCGGCGTCGGCGAAGTCGCCCGTCAGCGGGCG<br>GTCTTTACCGACCATATCACCATGATGGATGCGATAAACGGCGAAGAGGTCG<br>TGCACATTGAGACAGGACAGGCGTTCCGCGACCATTTTGGCGGGCCGTATGC<br>GGTGATCCACCGGGTGGATATCCATGCCACCGTCTGGGAGGCGGCGCTGACC<br>CATCCGGCCGTCGAGTATCGTACTTCGACGCAGGTAGTGGATATCCGCCAGA<br>CCGCCGATGACGTGACGGTGTTTGATGATAAAGGCAATAGCTGGACGGCGGA<br>TATTCTGATCGGCTGCGACGGCGGTAAATCGGTGGTGCGCCAAAGCCTGCTG<br>GGCGATTCGCCGCGGGTAACCGGCCACGTGGTCTATCGCGCGGTCGTCGATG<br>CCGCCGATATGCCGGACGATTTACGCATCAATGCGCCGGTACTGTGGGCGGG<br>TCCGCACTGCCACCTGGTGCATTATCCGCTGCGCGGCGGTAAGCAGTACAAC<br>CTGGTGGTGACCTTTCATAGTCGGCAGCAGGAAGAGTGGGGCGTGCGCGACG<br>GCAGTAAAGAAGAGGTGCTCTCCTACTTTAAAGGTATCCACCCGCGTCCCCG<br>GCAGATGCTCGATAAACCGACCTCCTGGCGCCGCTGGTCCACCGCCGATCGC<br>GAGCCGGTAGAAAAATGGGGCAACGATCGTATTACGCTGGTCGGCGATGCCG<br>CGCATCCGGTGGCGCAGTATATGGCGCAGGGGGCCTGTATGGCGCTGGAAGA<br>TGCGGTGACCCTGGGTAAAGCGCTGGCCCAGTGCGACGGCGACGCCGCCCGC

		GCTTTCGCGCTGTATGAATCGGTGCGTATTCCGCGTACCGCGCGGATCGTCTG<br>GTCGACGCGTGAAATGGGGCGGGTCTATCACGCTGCCGGGGTTGAGCGCCAG<br>GTGCGCAATCTGCTGTGGAAAGGCAAAACCCAGAGCGAGTTTTATCGCGGCA<br>TTGAGTGGCTGTACGGCTGGAAAGAGGGTAACTGCCTGCAACCACGTTAA
Klebsiella oxytoca BRL6-2_4-hydroxybenzoate 3-monooxygenase	WP_024273698.1	ATGAAAACACAGGTGGCGATTATCGGGGCTGGCCCATCCGGACTCCTGCTGG<br>GGCAGTTGTTACATCACGCCGGGATTCAAACCGTCATCCTTGAGCGACAGAC<br>TCCTGAGTACGTGCTGGGGAGGATCCGCGCGGGCATTCTGGAAAGCGGAACG<br>GTAGAGCTGCTGCGCGAAGCCGGCGCCGCCCGGCGAATGGAGGCTGAAGGC<br>CTGGTGCATCATGGCGTCGAATTTTTGTTTGAGGGCAAGCGGGTACCGGTTGC<br>GCTGACGGAACTGACGGCGGGCAAAAGCGTGATGGTTTATGGCCAGACTGA<br>AGTGACGCGGGATTTGATGGCCGCGCGGGCGGAATGCGGGGCGGCGACGAT<br>TTATGGCGTCAGCGAAGTCACTATTAATGAGGCAAAGAGCGATCGACCATCG<br>GTCTCTTTTATCAGCAATGGCGAACACTGCCGCTTAGAGTGCGACTTTATCGC<br>CGGCTGCGATGGCTTTCACGGCGTGTCCCGTCAAAGTATTCCTCCTGGGGTGC<br>TGAAAGAGTACGAAAGCGTCTGGCCGTTTGGCTGGCTGGGGCTGCTGGCCGA<br>TACGCCGCCGGTGAACCCGGAGCTTATTTACGCGCACCACGAACGCGGGTTT<br>GTTCTTTGTAGTCAGCGCTCACTAACTCGAAGCCGTTATTACCTCCAGGTCCC<br>GCTCAGTGAAAAGGTTGAGGCATGGTCTGATGAGCGTTTCTGGAATGAACTC<br>AAACGCCGCCTCCCTGAAGAGCTGTCCAGCAAGTTAGTGATTGGTCACTCAC<br>TCGAGAAGAGCATCGCGCCGCTGCGCAGCTATGTGGTGGAGCCGATGCAGTA<br>CGGCAGATTGTTTCTGGTGGGGGATGCGGCGCATATCGTACCGCCTACCGGG<br>GCAAAAGGATTGAATCTCGCGGCGTCCGATGTGAACTATTTATGGCGCATCCT<br>CCGTGAATATTATCACCATGGTCGCGTTGACCTGCTGGCGAGCTACTCTCGTT<br>TTGCGCTGGATAGGGTATGGAAAGGCGAAAGATTCAGCTGGTTTATGACCCG<br>TCTGCTGCATGATTTCCCGCAACAAAGCGAGTTCGATAGAAAAATGCAGGCG<br>GCGGATCGGCGTTATTATCTTGGCTCACGCGCCGGATTAACCACGATCGCTGA<br>AAACTATGTCGGTTTGCCGATGGAGCGCGTGGCATAA
Citrobacter sedlakii NBRC 105722_2-octaprenyl-6-methoxyphenyl hydroxylase	WP_042292169.1	ATGAGCGTGATTATCGTGGGCGGCGGCATGGTCGGCGCGACGCTGGCGCTGG<br>CTATTTCGCAACTCAGTCATGGGACGCTGCCGGTACATCTGATTGAAGCGACG<br>GCGCCGGAGGCCGATGGCCACCCCGGATTCGACGCCCGGGCGATAGCGCTG<br>GCAGCAGGTACCTGCCAGCAACTGGCGCGAATAGGCGTGTGGCAGGCCATTG<br>CCGACTGCGCGACCGCTATCACGAGCGTTCACGTCAGCGATCGCGGTCATGC<br>CGGCTTTGTTACCCTTCACGCTGAGGACTATCGTCTCCCGGCGCTGGGGCAGG<br>TGGTTGAACTGCACGACGTAGGGCTGCGGCTTTTTGCCCTGTTGCGCAAAGCG<br>TCTGGCGTTACGCTGCACTGCCCGGATCGGGTCGCCAGCGTATCGCGCACGG<br>CGGAGCAGGTGAGCGTGACGCTGGAAAACGGTCAGACGGTTACCGGTAGCC<br>TGCTGGTTGCCGCCGACGGCACGCGTTCAGCGCTGGCAAGCGCGTGTGGCGT<br>TGAGTGGCAGCAGGAACCGTATGAGCAACTGGCGGTGATAGCGAACGTCGC<br>CACGTCTCTTGCGCATGACGGCCGCGCCTTTGAACGCTTTACCGCGCATGGTC<br>CGCTGGCGATGCTGCCCATGTCTGAAGGCCGCTGTTCGCTGGTGTGGTGTCAC<br>CCGCTGGAAAAGCGCGACGAGATTCTGGCCTGGTCAGACGAACGTTTTTGCC<br>GCGAACTGCAGACAGCATTCGGCTGGCGGCTTGGCCGGATCACGCTGGCGGG<br>CAAACGTAATGCTTATCCGCTTGCGTTGACTACGGCGGCGAAAGCGTTTACCC<br>ATCGCACCGTACTGGTGGGCAATGCGGCGCAGACGCTGCATCCAATCGCCGG<br>ACAAGGGTTTAACCTCGGGCTGCGCGATGTGATGAGTCTTGCTGAAACCCTG<br>ACGCAGGCGCAGTCTGTGGGGCAGGATATCGGCGATTACAATGTGCTTTCCG<br>CCTATCAGCAGCGCCGCCAGCAAGACCGGAACGCCACGGTCGGCGTGACCG<br>ACGGCATGGTGCATCTGTTTGCCAATCGCTGGATACCGCTGGTGGTTGGCGC

		AACGTCGGGTTAATGGCTATGGAGCTGTTTCCACCCGCCCGTGACGCTCTGGT<br>GAAGCGCACGCTTGGCTGGGTTGCGCGATAA
Citrobacter sedlakii NBRC 105722_2-octaprenyl-3-methyl-6-methoxy-1,4-benzoquinol hydroxylase	WP_042285453.1	ATGACAAATCAACCAACGGAAGTGGCCATTGTCGGCGGAGGGATGGTCGGC<br>GGTGCGCTGGCGCTGGGACTGGCGCAGCACGGATTTACGGTCACGGTGATTG<br>AACACGCCGCGCCAGCGCCCTTTGCGGCGGACAGCCGGCCCGACGTGCGTAT<br>TTCCGCGATCAGCGCCGCGTCTGTTTCGCTGTTGAAAAGCCTGGGCGTCTGGG<br>ATGCGGTACAAGGGATGCGCAGCCATCCTTATCGTCGGCTGGAAACCTGGGA<br>ATGGGAAAATGCCCACGTTGTTTTGACGCTGCGGAACTGAAGCTGCCGCTG<br>CTGGGCTATATGGTCGAGAACACTGTTTTGCAGCAGGCGCTGTGGCAGGCGC<br>TGGAGGCGCATCCGAACGTTACCCTGCGAGTCCCAGCGTCGCTTGCGGCGCT<br>ACATCGTCATCACGACAGTCAGGTACTGGAGCTGGCAGATGGCGAGCAGATT<br>ATGGCGAAGCTGGTGGTCGGCGCTGACGGCGCGAATTCACAGGTGCGCCAAC<br>TGGCTGGCATTGGCATTCACGCCTGGCAGTATGCGCAGTCATGCATGCTGATC<br>AGCGTTCAGTGCGAACAGGCGCCGGGGGACAGCACCTGGCAGCAGTTTACG<br>CCGGATGGCCCGCGCGCCTTTTTGCCGCTCTTTGATCGCTGGGCGTCGCTGGT<br>GTGGTATGACGCGCCAGCGCGTATTCGCCAGTTGCAGACCCTGAGCATGCCG<br>CAGTTGCAGGCGGAAATCGTAAAACATTTCCCGGCGCGGCTGGGGCCGGTGA<br>CGCCGCTGGCCGCAGGTGCGTTCCCGCTGACCCGTCGACACGCGTTGCAGTA<br>TGTTCAGCCGGGCCTGGCGCTGGTTGGCGATGCGGCGCACACCATTCATCCGC<br>TGGCCGGGCAGGGGGTGAACCTCGGCTATCGCGATGTCGATGCGCTGATCGA<br>TGTGCTGGTAAATGCGCGCAGCTATGGAGAACGTTGGGCCAGCCAGCCGGTG<br>TTGAAGCGCTACCAAATGCGGCGCATGGCGGATAACTTTATGATGCAGAGTG<br>GAATGGATCTGTTTTACGCTGGATTCAGTAATAACCTGCCGCCGCTGCGTATG<br>CTGCGCAACCTCGGCCTGATGGCTGCTGAACGTGCGGGTGTTGTTAAACGCC<br>AGGCGCTGAAGTATGCGTTAGGGTTGTAA
Citrobacter sedlakii NBRC 105722_3-hydroxybenzoate 6-monooxygenase	WP_042284472.1	ATGGCTAAAGTGAAGAGCGCAATTATCGTTGGCGGCGGCATTGGCGGGGCAG<br>CAACCGCGCTTTCGCTGGCGCGCCAGGGTATCAAGGTGATGCTGCTGGAAAA<br>GGCGCACGAGATTGGCGAGATTGGCGCAGGCATCCAGCTTGGCCCAAATGCC<br>TTTTCGGCGCTGGACAGCCTCGGTGTTGGCGACGTAGCGCGCCAGCGGGCGG<br>TATTTACCGACCATATCACCATGATGGATGCGGTAAACGCGGAGGAAGTTGT<br>CCGCATTGAAACCGGCCAGGCGTTTCGCGACCATTTCGGCGGGCCGTATGCC<br>GTGATTCACCGCGTCGACATTCATGCCTCGGTCTGGGAAGCGGTGCTGACCCA<br>TCCTGATGTTGAATACCGGACATCCACCAATGTGGTGGATATTCGCCAGACGC<br>CGGACGACGTCACGGTATTTGATGAGCAGGGAAACAGCTGGACAGCCGATA<br>TTCTGGTGGGCTGTGACGGCGTGAAGTCGGTTGTGCGCCAGAGTCTGCTGGGG<br>GATTCCCCGCGGGTGACCGGACATGTGGTCTACCGGGCGGTGATTGACTGTG<br>ACGATATGCCGGAAGATTTGCGCATTAACGCGCCGGTACTGTGGGCGGGGCC<br>GCACTGTCACCTTGTTCACTATCCGCTACGCGGCGGCAAGCAGTACAACCTG<br>GTGGTGACGTTCCATAGCCGTCAGCAGGAGGAGTGGGGGGTGAAGGACGGC<br>AGTAAAGAAGAGGTGCTCTCGTATTTTGCCGGTATCCATCCTCGTCCACGTCA<br>GATGCTCGACAAACCCACCTCCTGGCGTCGCTGGTCCACCGCCGATCGCGAA<br>CCGGTGGAAAAATGGGGAACGGAGCGCATTACGCTGGTCGGTGACGCCGCG<br>CACCCGGTCGCGCAGTATATGGCCCAGGGCGCATGCATGGCGCTGGAAGATG<br>CGGTGACCCTCGGCAAAGCGCTGGAAAAATGCGAGGGCGATGCGGCAAGCG<br>CCTTTGCGCTGTATGAGTCGGTGCGCATTCCGCGCACGGCGCGAATTGTCTGG<br>TCGACCCGCGAAATGGGGCGCGTCTACCACGCTGCGGGCGTCGAACGTCAGG<br>TGCGTAATCTGCTGTGGAAAGGCAAATCGCAGGCGGAATTTTATCGCGGCAT<br>GGAGTGGTTATACGGCTGGAAAGAGGATAACTGTCTGCTGCCGCGCTAG

Alcaligenes sp. Strain HPC1271 _aromatic-ring-hydroxylating dioxygenase subunit beta	WP_009463815.1	ATGAGCGAAATCAGCCGTCAGGCCCTTATCGATTTTGTGTATGCGGAAGCCC<br>GCCTGCTGGATGAGCAGCGCTACGAGCAATGGCTCGACCTGTTCACCGAGGA<br>TGGCTACTACTGGATGCCGCTGGTGCACGATCAGCAGGACGCCCGTTTGCAC<br>GCGTCCCTGATGCACGAGGACAAGCTCTTGCTGCGTATCCGTGTGGAACGTCT<br>GGCAGGCCGCCGTACCTTTTCCCAGCAGCCCAAGAGCCGCAGCCATCACCTG<br>CTCCAGCAACCCACTGTGGAAAGCATGGACGAGGAAAAAGGCGAATACACC<br>GTGCGTTGCGCCTTCCATTACACGGAAACGCGTGGCGACAACCAAGATATCT<br>ATGTGGGTTGGAATACCTACACCCTGGTGCGCCAGGACGACGCCTTGAAGAT<br>CCGTCTGAAACGGGTGGATCTGCTCAATTGCGACGCGCCTTTCGGCAATATCC<br>AGTTGTTTATGTGA
Alcaligenes sp. Strain HPC1271_monooxygenase	WP_003800437.1	ATGACATCCAAGGTTTATATCGGCCTGCAAGACAACGACATGTCGCGCTACA<br>TCGTCGAGGCCATCGAGGAAGACAACCCGGACGCGACGGTGATTTATCAGCC<br>AGCCATGATCCGTATTGAGTGTGTGGGTCAGTTGACCGTCAAGCGCGAGACC<br>GTGGAAGAAAAGTATGGGCAGCAATGGGACATGCAGGAGCTGCATCTGAAT<br>CTGATCACCTTGGGTGGCAACGTGGATGAAGATGAGGACCGCTTGTTTTTGCA<br>CTGGAACAGCTAA
Alcaligenes sp. Strain HPC1271_phenol 2-monooxygenase	WP_009454452.1	ATGTCAGACACGAGCTTGCCCACTTTGACAAAGTACGTACGCGTACGCAGTC<br>CGGCCAACGCCCGTTTTGTGGAATTTGATTTTGCCATCGGCCAGCCGGACCTG<br>TTTGTGGAACTGGTCATGCCCCCGGCGGCGTTTGAGCAGTTTTGTCTGCAAAA<br>CAATGTCCAGCCCATGAGCGATGAGCAAATGCGGGTCAATGACGAGAACGA<br>AGAGAAATGGCGTTTTGGTTATGACACTTTGGTCGGCAACAGTCGTCAGGCTG<br>AAGCCCAGTAG
Alcaligenes sp. Strain HPC1271_phenol hydroxylase	WP_009454448.1	ATGGCAGTTACTGCAATTACCCCGGACTACCAAGGCTATGCGCGCGACCAGC<br>AGTCGCACTATGGCGACAGCATGTTGCTGTATATAGGCTGGGACGAGCACCT<br>TTTGTTCTGCTCGGCCAAGGCCTTTCTGGTTCAGCCCGAGATGACCGTGTCCC<br>AGATGATCCAGCAACTGTTGCCTGCCGGTTTTGCCCAGCACCCTGACTTCGAG<br>CAGATCGAATGGAGCAAGGTGCAATGGAATTTGAACGGCGAGGCGGTTGAA<br>CTGAATCCTGACGACACCTTGGGTTCCAAGGGCTTCGATCACAAATCCTTGTT<br>GCGTTTCAAGACCCCTGGCCTGAATGGCTACAAGGGTACGGGCGTATAA
Alcaligenes sp. Strain HPC1271_cytochrome P450	WP_009457883.1	ATGTTTGGCGATTTTGATCTTGCGTCACCCTATGCAGCTTATGCCCGTCTTCGT<br>CAGGGGCCGCCTTTGGTTTGGGATGCGGATTTCTGCGGTGGGGCGTGGTTAGT<br>TCATAGGCACAGCGATGTACAAGCCGCCCTGCGCAATCCCGATTTAAGTGTG<br>CGGCGTGTTGGCGGGTGGGTGGCCCAGGCTGGCGCTGACCCCTCCCCTCGTCT<br>GCAAGCCTTCAAGGGCTTGATGGCCAGGACGCTGGTCTTTCTGGACCGCCCC<br>CGTCACACACGCGTCAGACGAGTAGTGAACGCGGCTTTTAGTGCGAAGGCTT<br>TGGAAACCCTGCACAGCACGATTGAAAAGCGCGTTACGCATATCCACCTGGA<br>GTTAAAAGACAAGCTGCGGCACGGTTCAGTGGATCTGGTTGATGAGGTCTGT<br>CGCCCACTCCCCGCCTTGGTCATGATGGACGTGCTCGGCTTGGAGCAACTGCC<br>TTTGTCTGTTTTTCAGGCCTGGTCAGAGCAACTGGCAGGTTTTATCGGACAAG<br>CCACGCCGGATATGCCTTTGCTGAGAAGCACCCAGGACGCCTTGCTGGATAT<br>GGCTGACTTTCTAGGCAACAGTGCCAATCTGCAAGAAGGCGGGTTGGCCTGG<br>CGGCTTTTGCATGATGAGCAGCTTTCCCTTCAAGGCAGGCGCGAGCGCTTGGC<br>ACAGTCCTGTATGTTGCTCTTTGCAGGCTATGAAACCTCCCGGCATCTGCTTA<br>GCTCCCTGTTTCAAACACTGCTGGCCGACTCCACAACGCTGCAGGACTTGCTA<br>TCGAATCCGCAGAAAATTCCTGCCGCGATCAAAGAGGTGCTGCGTCACGACA<br>GCCCGATTCAGTACACAGCCAGACGTTTGATGCGCGACCAGCACTGGCATGG<br>TCAGCATTTGCGCAAGGGGCAGTTGCTGCTTTTGCTCTTGGGCGCTGCCAACC<br>GCGATCCGGCGGTGTTTGCCAATCCCGACGATCTGGACTTCAGCCGCAACAA

Alcaligenes sp. II-C-7_alkane hydroxylase	ACJ22769.1	TCAGGCCGAGCTATCCATGGGACACGGAATTCATCATTGCCTGGGAGCCGGT<br>TTGGTGCAGCTGGAAGCCAAGCTCGTACTTGAGCAATTCCTGCCTTTGCTCCC<br>TCGCCTGCAAGGGACGCAAGGTGAGCGTATTCGACTCCCCGCCTATCGGGGC<br>TGGAACCGCTTGCCGGTTCAATACCGGGCGGCACAGTCATGA
Aeromonas taiwanensis LMG 24683_2-octaprenyl-6-methoxyphenyl hydroxylase	WP_043759840.1	AATACCGGCCATGAGCTGGGGCACAAGACCGACCGCCACGAGAAATGGATG<br>GCCAAGCTATGTCTCGCCCCGGTGTTCTATGGGCACTTCTATGTGGAACATAA<br>TCGTGGGCATCACGTGAGGGTTTCCACGCCGGAGGATCCGGCATCCTCCCGG<br>TTCGGCGAGACGTTCTGGGAGTTCCTGCCGCGCACCGTGATCGGCAGCCTTAA<br>ATCCGCCTGGTCGCTGGAGAAACAGCGGCTGGAGCGGCAGGGGCTTTCGGTA<br>TGGAGCTGGCATAACGACAATCTGCAGGCCTGGGCATTGTCGGTGGTGCTTTG<br>GGGTGCCCTGATACTGTGGTTGGGCTGGGCGGTGGTGCCGTTCCTGCTGATTC<br>AGAGCCTGTTCGGTTTTCAGTTGCTGGAGGTGGTCAACTATATCGAGCA<br>ATGTCTCAATCTGATCTGGCTCACGTTGACGTGACGATCGTCGGCGGCGGTAT<br>GAGCGGTGCCGTGCTGGCCCTCTCCCTCGCCGCCCTCCGGGGATCGGACGGT<br>GCCCCCCTGCAGATCCTGCTGCTGGAGGCGAGCGCCCCCGAGCTCAATGCTC<br>ATCCCGGCTTCGACGCCCGCGCCATCGCCCTCTCGGCGGGCACCTGCGAGGC<br>GCTGACCCGCCACGGCATCTGGTCCCGTTTCACCCCCCATTGCACCCCCATCA<br>CCGACATTCACGTCTCGGATCGGGGCCACTGCGGCCAGGCCAGGCTGTCGGC<br>GGCGGAATATGGCTTGCCGGCGCTCGGCCAGGTGATCGAACTCTCTGCCGCC<br>GGGATCGAGTTGCAGCGGGCCCTTGCCGCCACCCCGCAGATACGGGTGTGCT<br>GCCCGGCCCAGCTCGCACAGATTGAGCCCGACGAAGAAGGGGTGACCCTGA<br>CCCTCGCCACGAACGAGCGCTATCGGACCCAGCTGCTGGTGGCGGCGGATGG<br>GGGCAACTCCTTCGTGCGCCAGCACTTCAAGATGCCGGTCAGTCGCCACGAC<br>TATGGCCAGAGCGCCATCATCGCCACGGTGAAGACCGCCCTGGACCCCGCCG<br>GGCGGGCCTTCGAGCGCTTCACCGAAGGGGGGCCGCTCGCCCTGCTGCCGAT<br>GCAGGAGGGGCTCTCCTCCCTGGTCTGGAGCCTGGCGCGGGACGAGGCACAG<br>GCCCTGATGGCGCTGGACGACGCGGCGTTTCTCGCCCGCCTGCAGCAAGCCT<br>TTGGCTGGCGCCTCGGGCGTTTCGAACGCACCGGCGTGCGCCACCTCTATCCG<br>CTGGTGCTGACGGTGGCCGATTATCCCCTGGCCCAGCGCACCGTGCTGGTGG<br>GCAACGCCGCCCATCTGCTGCACCCCATCGCCGGTCAGGGCTTCAACCTCGG<br>CATGCGCGACCTTGACCTGCTCACCCGCGCCGTTGGCCAGGCGCTCCTGGCG<br>GGGGAGGACATCGGCAGCTTCGAGGTGCTGAACGGTTACTGGCAATCGCGCC<br>GGGAGGATCAAGCCCAGACCGTCTGGCTCACCTCCTCCCTGGCCCAGCTCTTC<br>TCCAACGGCCACGCTCCCCTGGTGGCGGGGCGCAACCTGGCGCTCTCTCTGAT<br>GGGGCGACTGCCCTGGCTCAAGGCGCCGCTGGCTTCCCGCACCCTGGGGTTC<br>GTCACCGACGTGTGCCGGCCATGA
Brevundimonas diminuta 3F5N_monooxygenase	WP_087139175.1	ATGATCAAGCCCGTTTCCATCAGCATTCAGAACACCCAGGACGGTTTCGCCAT<br>CATCGAAGCCATCCTGGCCGACAACCCCGAGGCCAAGAGCAACCCCTTCCCG<br>GCCATGACCAAGATCGACTGCCCCGGTCGGCTGGAAATCCGCGCCGAGAGC<br>GTTTCCGAACGCCTGGGCCGCGACTGGGATCCTCAGGAAATCCACCTCAGCG<br>TCATCTCCCTGTCCGGTTCGGTGGACGAGGACGACGGCTCTCTGATCATCTTC<br>TGGAGATAG
Brevundimonas diminuta BZC3_phenol hydroxylase	WP_088410947.1	ATGAACATCGACATCCAGGCCGAGGCGATCACGCCGCGGCGTCATACCTTCT<br>CGCACATCGCGCGGCGCTTCGGCGTCGACAAACCCGCCTCGCGCTATGAAGA<br>GGCGACCTATGACGTGCAGCCGGTGGTCAACTTCCACTATCGGCCGACCTAT<br>ACGCCGCAGTTCGAACTGTACGACCCGCGCCGCACCCGCATCGTGATGAACG<br>ACTGGTACGTCTTCCGCGATCCGCGCCAGTTCTATTACGCCTCCTACAACATC<br>AGCCGCGCGGCCATGCAGCAGTCGTTCGACGACGCCGTGAACTTCGTCGAGA

		AACGCGACCTGATCGCCGATGTCGCCCCGGAATGGCGCGACCTGTTCGCCGC<br>CTGGCTGCTGCCGCTGCGCCATGTGGAATGGGCGGCGAACATGAACTGCCAG<br>CTGATCGCCGACTGGGGCTATGGGACGCAGATCACCTCGGCGGCGGCCTTCT<br>GCGGGGCCGACCGCCTTGGCATCGCCCAGGTCATCTCGCGCATCGGCCTGCT<br>GCTGGGTCAGGGCACGGAAAGCGCCCTGAAGGAAGCCAAGACCCAATGGCT<br>GGAGGCCGACGTCTGGCAACCCCTGCGCCGCCTGGCCGAAGACCAGCTGGTC<br>GTCCATGACTGGTTCGAGCTCTATGTCGCCCAGCTGATCGTGCTGGACGGCTA<br>CATCTATCCGCTGGCGACGGGCGCCTTCGACCGTGCGGGCGCGGCGCACAAC<br>GGCGCCGCCTTCTCGATGATGACCGGCTTCCTGGGCGACTGGTACGCCGACA<br>ACAGCCGCTGGACCGACGCCGTCATCAAGGCCGCCGCCGCAGAGTCCGAGG<br>CCAACCGCATCGTTTTGGGCGAATGGTTCGAGCGCTGGTCCGCCCGCGCCGA<br>AGAAGCCGTCCGCCCCCTCGCCGCCATGGTGTTGGGCGCCGAAGGCGACGCC<br>GCCGTCGATGCGGTCGGCGCGCGCCTGCGTGAACGGATGGTCGGACTGAACG<br>CCCTAGAAGCGCGGGAGATGGCGGCATGA
Brevundimonas diminuta NCTC11164_3-(3-hydroxyphenyl)propionate hydroxylase	SPU44975.1	ATGCTTGATTCCATGAACGACGCCGCCGCCTCCCTCCCCGTGGTCATCGTCGG<br>CGGCGGGTTCTCCGGCGCCATGCTGGCCGTGCGCCTGGCCGAACTGGGCCAA<br>GCCTCGGTCCTGATCGAGCGCAGCGAACAGGTCGGCCTCGGCGTCGCCTATT<br>CCACGCCGCTGGACGACCACCGGCTGAACGTCCGCGCCGAGCGGATGAGCG<br>CCCGCCCCGACCGGCCCGCCGACTTCACCGACTGGTTGGCGCTCCACGCCCC<br>CGCCTTCGCCGATCCGAACGGCTTCGCCCCGCGCCGCCTCTACGGCCTCTATG<br>TGCAGGCCCGCCTGGCCGAGGCCGAGGCCGCCCGTCCCGGCCTGATCCGTCG<br>TGTGAAGGGCGAGGGCGTCCGCATCGAAGGCGAGACCGTCCTCCTCGCCGAC<br>GGCGCCCGCATCCCCGGCCGCGCCGTGGCGCTGGCCACCGGCAACCCGCCGC<br>CGCGCCCCGCCGTGGCGGGCGAAGGCCCCCGCCGCATCGCCGACCCGTGGC<br>GGTCCGACGCTCTGGCCGCGATCGGCCCCGACGATGACGTGCTGATCCTCGG<br>TTCCGGCCTGACCATGGTCGATGTCGTTCTGGCGCTTCAGGCTCAGGGCTGGC<br>GCGGCCGGGCGACGGTCGTGTCGCGGCGCGGCCTGCTGCCCCAGGCCCACGG<br>CGCCCATCACGACGCCCCCATCGACCTGCCGCCCGAGGCGCTGACCGGCGCC<br>CTGTCGCGCCGCCTGGCCTCGGCGCGACGACTGGCGCGCGATCACGGCTGGC<br>GCCGCGTCATGGAGGGCTATCGCCCCGTCACCGCCGCGCTGTGGGGCGCCGC<br>GACGATCGCCGAGCGCGGCCGCTTCCTGCGCCATCTGCGTCCCTGGTGGGAC<br>GTTCATCGCCACCGCATCGCGCCCGAGATCGCCGCCGTGCTGGAAAGGTTGA<br>AAGCGGAGAACCGACTGACCGTCGTCGCGGGCCGCGCCCAGGCGATGGCGG<br>CCGACCGCCAGGCCGCGAACCTGACCGTCCGTCCACGGAACGGCGAAGTTC<br>AAGCCTTCTCCGCCGCCTGGTTGATCGACTGCACCGGTCCCGGCCACGACGC<br>CGCCTGCACGCCCCTGACCGCCGCCCTGATCGCCGAAGGCCGCGCCCGCCTC<br>GATGCGGTCGATCTGGGCCTCGACCTCGACGCCGAGGGCCGCGTGCTGCACG<br>CCGACGGCGTCGCCGACCCCGGCCTGTTCGTGCTCGGCCCCCCGGCTCGCGC<br>CGCCTTCTGGGAGACGATCGCCGTGCCGGACATCCGCCAGCGCATCGAAGCC<br>CTGGCCCGCGCTCTCGCCGATTGGCGCCAGCCGTTCAGGCGCGGTCGGAATTT<br>CTAA
Serratia marcescens subsp. marcescens Db11_2-octaprenyl-3-methyl-6-	WP_025301854.1	ATGAAGACATCTCAAAATCGGTATGACGCGGTGGTGGTGGGTGGCGGCATGG<br>TCGGCGCGGCGGCGGCCCTGGGGCTGGCGCAGGCGGGCTGGTCGGTGGCGTT<br>GCTGGAACATCAGGCGCCGCAGGCGTTCGAGGCGCAAAGCCCGCCGGACCT<br>GCGCATTTCCGCGATCGGCTGCACCTCGGTGGGGCTGCTGAAACAGCTCGGC<br>ACCTGGCAGGCCGTGACGGCGATGCGTACGGCGCCGTATCGCCGGCTGGAGA<br>CCTGGGAGTGGGCGTCGTCGTGCGTGGCGTTCGACGCGGTGTCCCTGGGGCTG<br>CCCGAGCTGGGCTTTATGGTGGAAAACCGCATTCTGCAGCTGGGGCTGTGGC

methoxy-1,4-benzoquinol hydroxylase		AACAGTTCGCGCAGTGCGCCAACCTGACGCTGCTGTGCCCCGCCAGGCTGCA<br>ATCGCTGCAGCGGGCGGACAATGCCTGGCAGCTGACGCTGGACAGCGGCGA<br>GACGCTGCAAGCGCGGCTGGTTGTCGGTGCCGACGGGGCCAACTCGCAGGTG<br>CGCAAGCTGGCGGCGATCGGCACCAACGGCTGGCAATATCGTCAGGCGTGCA<br>TGTTGATCACCGTCGATACCGGCGCGCCGCAGCAGGATGTGACCTGGCAGCG<br>CTTCTTCCCGTCCGGGCCCCGCGCCTTCCTGCCGCTGTACGACAGCTGGGCGT<br>CGCTGGTGTGGTATGACAGCCCGCAGCGCATTCGCCAACTGCAGGCGATGCC<br>GCCGGCGCAGCTGGAGCGCGAAATCGCTACCGCTTTTCCGGCGCGACTGGGC<br>CCGGTCAAGGTGCACGCTGCCGGATCGTTCCCGCTGACGCGGCGTCATGCGC<br>AGCGCTACGTGCTGCCGGGGCTGGCGCTGGTGGGCGATGCGGCGCATACCAT<br>CAACCCGCTGGCCGGGCAGGGCGTCAATCTGGGGTATCGCGATGTGGATGCC<br>TTGCTGAACGTGCTGAGCGAGGCGCGGGATCAGGGAGAGGACTGGAGCAGC<br>GAGGCGGTACTGCTGCGTTATCAGCGTCGCCGCCGTACCGACAATCTGCTGAT<br>GCAGAGCGGCATGGATCTGTTTTATACCGCCTTCAGCAATAACCTGGCGCCG<br>CTGAACGTCGCGCGCAATCTGGCGCTGATGGCCGCGCAGCGGGCGGGCAAG<br>CTGAAAGAACATGCGTTGAAGTACGCGTTGGGATTGTAA
Pseudomonas aeruginosa DSM 50071 = NBRC 12689_4-hydroxybenzoate 3-monooxygenase	AKO84441.1	ATGAAGACTCAAGTCGCCATCATCGGCGCCGGTCCGTCCGGCCTCCTGCTCG<br>GCCAGTTGCTGCACAAGGCCGGCATCGACAACGTGATCCTCGAACGCCAGAC<br>CCCGGACTACGTGCTCGGCCGCATCCGCGCCGGCGTGCTGGAACAGGGTATG<br>GTCGACCTGCTGCGCGAGGCCGGCGTCGACCGGCGCATGGCGCGCGACGGG<br>CTGGTCCACGAAGGCGTGGAGATCGCCTTCGCCGGGCAGCGCCGGCGCATCG<br>ACCTGAAGCGCCTGAGCGGCGGCAAGACGGTGATGGTCTACGGCCAGACCG<br>AGGTCACCCGCGACCTCATGGAGGCCCGCGAAGCCTGCGGCGCCACTACCGT<br>CTACCAGGCCGCCGAGGTGCGCCTGCACGACCTGCAAGGTGAGCGCCCCTAC<br>GTGACCTTCGAACGCGACGGCGAACGGCTGCGCCTGGATTGCGACTACATCG<br>CCGGCTGCGATGGCTTCCACGGCATCTCGCGGCAATCGATCCCGGCGGAACG<br>GCTGAAGGTCTTCGAGCGGGTCTATCCGTTCGGCTGGCTCGGCCTGCTCGCCG<br>ACACCCCGCCGGTCAGCCACGAACTGATCTACGCCAACCATCCGCGCGGCTT<br>CGCCCTGTGCAGCCAGCGTTCGGCGACCCGCAGCCGCTACTACGTACAGGTG<br>CCATTGACAGAGAAGGTCGAGGACTGGTCCGACGAGCGCTTCTGGACGGAA<br>CTGAAAGCGCGCCTCCCGGCCGAGGTGGCGGAGAAACTGGTGACCGGTCCTT<br>CGCTGGAGAAGAGCATCGCGCCGCTGCGCAGCTTCGTGGTCGAGCCGATGCA<br>GCATGGCCGGCTGTTCCTCGCCGGCGACGCCGCGCACATCGTGCCGCCCACC<br>GGCGCCAAGGGACTGAACCTGGCGGCCAGCGACGTCAGCACGCTCTACCGG<br>CTGCTGCTGAAGGCCTACCGCGAAGGGCGCGGCGAACTGCTGGAACGCTACT<br>CGGCAATCTGCCTGCGGCGGATCTGGAAGGCCGAACGCTTCTCCTGGTGGAT<br>GACTTCGGTGCTGCATCGCTTCCCCGACACCGACGCGTTCAGCCAGCGCATCC<br>AGCAGACCGAACTGGAGTACTACCTGGGCTCCGAGGCGGGCCTGGCGACCAT<br>AGCCGAGAACTATGTCGGCCTGCCCTACGAGGAAATCGAGTAG
Pseudomonas aeruginosa DSM 50071 = NBRC 12689_cytochrome P450	WP_003091710.1	GTGCCTGATCGCAAACTGAGACTGGGCGAGGAACTGATCTCGCCACTGCACG<br>CGCTCTACGACGGCCTGCAGGTGGACGGCGCGCCGCGTCCCGCGCATCGCGC<br>CGCCGAGCATCCGGTGTGGGTGGTGACGCGCTACCGCGACGCGCGCAAGGTC<br>CTCAACCATCCGGGCGTCCGCCGCGACGCCCGGCAGGCCGCCGAACTCTACG<br>CGAAGCGTACCGGCAGCCCGCGCGCGGGGATCGGTGAGGGACTCAGCCACC<br>ACATGCTCAACCTCGACCCGCCGGACCATACCCGCCTGCGCTCGCTGGTTGG<br>CCGCGCGTTCACCCCGCGCCAGGTGGAGCGCCTGCAACCGCATATAGAACGG<br>ATCACCGAGGAATTGCTGGACGCCATGGCCGGCCGCGAACAGGCCGACCTG<br>ATGGCCGACTTCGCGATCCCGCTGACCATCGCGGTGATCTTCGAGCTGCTGGG

		CATTCCCGAGGCCGAGCGCGAACACGCCCGCCAGTCCTGGGAGCGCCAGGC<br>GGAACTGCTGTCGCCGGAGGAGGCCCAGGCCCTGGCCGATGCGCAGGTCGA<br>CTACCTGCGCGTGCTGCTCGAGGCCAAGCGCCGGCAGCCGGCCGACGACGTC<br>TACAGCGGGCTGGTGCAGGCCGCCGACGAGAGCGGCCAGTTGAGCGAAGCG<br>GAACTCGTCTCCATGGCCCACCTGCTGATGATGAGCGGCTTCGAGACCACCA<br>TGAACATGATCGGCAACGCGCTGGTCACCCTGCTGGTCAACCCGGAGCAACT<br>GGCGTTGCTGCGGGCGCAGCCGGAACTCCTGCCCAACGCCATGGAAGAACTG<br>GTCCGCCACGACAGTCCGGTGCGCGCCTCGATGTTGCGCTTCACCGTGGAAG<br>ACGTGGAACTGGACGGGGTCACCATTCCCGCCGGCGAATACATCCTGGTCTC<br>CAACCTGACCGCCAACCACGATGCCGAGCGCTTCGACGATCCCGACCGCCTC<br>GACCTCACCCGCAACACCGATGGCCATCTCGGCTACGGCTTCGGCGTGCACT<br>ACTGCGTCGGCGCCTCGCTGGCCCGGCTGGAGGGGCGGATCGCCATCCAGCG<br>CCTGCTCGCGCGCTTCCCCGACCTCCAGTTGGCGGTGCCCCACGCGGAGCTGC<br>AGTGGCTGCCGATCACCTTCCTCCGCGCCCTGATCAGCGTGCCGGTGCGCACC<br>GGATGCAGCGCCCCGGCGAACACCGCCTCCCACGCCAACCCGATCGAGAGG<br>ATCGCCCAATGA
Pseudomonas aeruginosa DSM 50071 = NBRC 12689_2-octaprenyl-3-methyl-6-methoxy-1,4-benzoquinol hydroxylase	WP_003120388.1	ATGCACGCGGATCTGGTCATCGTCGGCGCCGGCATGGTCGGTAGCGCCCTGG<br>CCCTGGCCCTGGAGGGCAGCGGCCTGGAGGTGCTGCTGGTGGACGGCGGCTC<br>GCTCGACGTGGCGCCGTTCAAGCCGGAGGCGCCCTACGAGCCGCGGGTCAGC<br>GCGCTGTCCGAAGCCAGCCGGCGGATTCTCCAGCGCCTGCATGCCTGGGACG<br>GCATCGTCGCGCGCCGCGCCGAGCCCTATCGCGAGATGCAGGTGTGGGATGG<br>CTCCGGCACCGGGCGGATCGGCTTCAGCGCCGCCAGCGTGCACGCCGAGGTA<br>CTCGGCCATATCGTCGAGAACCGGGTGGTGCAGGACGCCCTGCTGGAACGCC<br>TGCACGATTCCACCACCGGCCTGCTGGCCAATGCGCGCCTGGAACAGATGCG<br>CCACTCCGGCGACGACTGGCTGCTGACCCTGGCCGACGGTCGCCAGCTGCGT<br>GCGCCGCTGGTGGTCGCCGCCGACGGCGCCAACTCGGCGGTACGCCGCCTGG<br>CCGGCTGCGCCACCCGCGAATGGGATTACCTGCACCACGCCATCGTCACCAG<br>CGTGCGCTGCGAGAACGCGCACCGCGCAACCGCCTGGCAGCGCTTCACCGAC<br>GACGGTCCGCTGGCCTTCCTGCCCCTGCCGGACCGCGGCGACGAGCACTGGT<br>GCTCGATCGTCTGGTCGACCACCCCGGAACAGGCAGAACGCCTGATGGCGTT<br>GGACGAGGATGGCTTCCGCGCCGCCCTCGGCGAAGCCTTCGAACAGCGCCTG<br>GGCGCCATCCTGCATGCCGACCGGCGCCTGTGCATCCCGCTGCGCCAGCGGC<br>ACGCCAAGCGTTACGTCGAGCCGGGCCTGGCGCTGGTCGGCGATGCTGCGCA<br>CACCATCCACCCGCTGGCCGGACAGGGCGTGAACCTGGGCTTCCTCGATGCG<br>GCGGTGCTGTCCGAGGTCCTGTTGCACGCCCTGGAGCGCGGCGAACGGCTTG<br>CCGACGAACGCGTGCTGAGTCGCTTCGAGCGGCGGCGGATGCCGCACAACCT<br>GGGCATGATGACGGCGATGGAAGGCTTCGAGCGCCTGTTCCAGGCCGACCGG<br>TTGCCGCTGCGCTGGCTGCGCAACGCCGGCCTGCGCCTGGTCGACGGCCATC<br>ACGAGGCGAAGGCGCTGTTCGTCCGCCAGGCGCTTGGCCTCAGCGGCGACCT<br>GCCGTCGCTGGCACGAGTCTGA
Pseudomonas aeruginosa DSM 50071 = NBRC 12689_alkane 1-monooxygenase	WP_003083349.1	ATGTTTGCCTCGCTTTCCTCTGCCTGGATGCTGCGTCTGAAAAAGTACGGCTA<br>CTGGATCTGGCTGATCGCGGTGCTCGGCATCCCGCTCAGCTACTGGTGGTCGC<br>TCGGTAGCGACTACCCCAACGCCTGGCCCTGGCTGGTGATCAGCGTGGTGTTC<br>GGGCTGATCCCGATCCTCGATGCCATCGTCGGCCGCGATCCGGCCAACCCCG<br>AGGAAGCCAGCGAAGTGCCGGAGATGGAAGCACAGGGCTACTACCGCGTAC<br>TGTCCCTGGCCACCGTCCCGCTGTTGCTGGGCATGCTCGTCTGGTCCGGCTGG<br>ATCCTCGCCCACGAGACCCGCTGGGACTGGGTCGGCCAACTGGGCTGGATCC<br>TGTCGGTGGGCACCGTGATGGGCGCCATCGGCATCACCGTCTCCCACGAACT

		GATCCACAAGGACCCGCAACTGGAACAGAACGCCGGCGGCCTGCTGCTGGC<br>AGCGGTGTGCTATGCCGGCTTCAAGGTCGAACACGTGCGCGGCCACCATGTA<br>CACGTCTCGACCCCGGAAGATGCCTCGTCCTCGCGCTACGGCCAGAGCCTCT<br>ACTCGTTCCTCCCGCACGCCTACAAGCACAACTTCCTCAACGCCTGGCGCCTG<br>GAGGCCGAGCGCCTGAAGCGCAAGGGCCTGCCGGCCCTGCACTGGCGCAAC<br>GAGCTGATCTGGTGGTACGCCATCAGCGCCCTCTTCCTGCTCGGCTTCAGCCT<br>GGCCTTCGGCTGGCTGGGAGCGATCTTCTTCCTCGGCCAGTCGGTGATGGCCT<br>TCACCCTGCTGGAGATCGTCAACTACGTCGAGCACTACGGCCTGCATCGGCG<br>GCGCCTGGACAACGGCCGCTACGAACGCACCACGCCGGAACACTCGTGGAA<br>CAGCAATTTCCTCCTGACCAACCTGTTCCTTTTCCACCTGCAGCGCCATTCCGA<br>CCACCATGCCTACGCCAAGCGCCGCTACCAGGTGCTGCGCCACTACGACAGC<br>AGCCCGCAACTGCCCAACGGCTACGCCGGGATGATCGTCCTCGCCCTGTTCC<br>CACCGCTCTGGCGCGCGGTGATGGACCCGAAGGTGCGCGCCTACTATGCCGG<br>CGAGGAATACCAGCTTACCGACACCCAGCGCATCTGA
Rhodococcus ruber OA1_hydroxylase	RQM33257.1	ATGGGAGCCGAAACGTCACCGTCACCGTCCCGTCCATCCGCCGCCCCGGTGC<br>GCGTGCCGGCCGACCATCCCGGCGTCGCCGAGCTGTTCGCGGTGCTCGCCTA<br>CGGTGAGATCTCGGCCTTCTATCGCCTTGCCGACGATGCGCGGATGTCGCCGA<br>GCCTGCAGGGGCGGGTGGCGTTCGCAAGCATGGCGGCGGCGGAGATGAGCC<br>ACTTCGAGACGCTGCACGCCGCCCTGACCGCGCGCGGTGTCGAAATCTACGC<br>GGCGATGGAGCCCTACCGGCGTGCCCTCGACGCCTACCACGCGTCCACGAAC<br>CCGTCGAACTGGCACGAGTCGCTGGTCAAGGCATATGTCGGCGACGGCATCG<br>CCGCCGACTTCTACCGCGAGATCGCCGGTACCCTCGCCCCCGAGGTGGCCGA<br>GGTCGTACGAGAGGTGCTCGCCGAGACCGGGCACTCGGAATTCGTGGTGCAC<br>GAGGTCCGCGAGGCGGTGCGGCACAGCGCCGCCGACAAGGACCGGCTGATG<br>CTGTGGGGGCGGCGGCTGCTGGGCGAGGCCATCACCCAGGCCCAGTACGTGA<br>TGGCCCAGCGGGAGGATCTCACCGATCTGGTGATCGCCGCTACCGGTGATCT<br>CGCCGACATCGCGGCGTTGTTCGACCGGATGCAGCTCGAGCACGCCGACCGG<br>ATGGCAGTGCTGGGACTGCACTAA
Rhodococcus ruber YC-YT1_aromatic ring hydroxylase	WP_119699570.1	ATGTCGACCTACTACCAGCCCCGCAAGTACCCCGCCTCCGACTTCGCGTCGAT<br>GCAACCCGACGCCGAGGCGCTGCCCGTGGTCGTCGTCGGAGCCGGACCCGTC<br>GGCATGGCCGTCGCCCTCGGCCTCGCCCAGCGCGGCATCCCCGTGACGATCC<br>TCGAGGCGGCAGTCCAGGTCTCCTTCGGCAGCCGTGCCATCTGCATCTCCCGG<br>CACAGCCTCGAGGCCGCCGCACGTCTCGGCTTCGGCGCCGAGCTGGAGAAGA<br>TCGTCCTGCCCTGGGTCGGCGGCCGCAGCTTCTACCGTGACCAGCAGGTGCTG<br>CACTTCCGGATGCCGCAGCGCGAGTTCGACGTGCGACCGCCGATGATCAATG<br>TCTCGCAGTCCGAGTACGAGCAGATCGTCGTCGACACCATCGAGCAGAACCC<br>GCTGATCACCCTGCACTGGCAGGCGAGCATCGCCGGCATCCTGCGCGACGAC<br>GACCACGTGACCCTGGAGGTCGACACCGCATTCGGCAAGCGTCACCTGCGGG<br>CCGCATGGGTCGTCGCCGCCGACGGCGGCCGCAGCCGCATGCGTGAGCTCGC<br>CGGTCTGCGACTCGAGGGCACCAACTACGAGGGCCGCTACGTCATCGCCGAC<br>ATCCACTGGGAGTCGGAGCTGCCCGCCGAGCGCATGGTCTGGTTCGATCCGC<br>CGAGCAACCCCGGCTCGACGATCATCATGCACAAGCAGCCGAAGAACATCT<br>GGCGCATCGACTACCAGCTCGATCCGTCCGAGGACGCGGAGATCGAGACCC<br>GCGAGGACGCCATCCGGGCCCGCATCACCAAGCACCTGGACTGGCTGCAGA<br>ACGACCTGCCCTGGACGCTCGAGTGGCACGGGTTCTACAGCGCCCGCGCCCT<br>CGCGCTGCGCGAGTTCACCCACGACCGCCTCCTCTTCGCCGGCGACGCCGCG<br>CACCTCGTGCCGATCTTCGGCGTGCGCGGCCTGAACTCCGGCATGGAGGACG<br>CCGAGACCCTGGCCTGGCAGCTCGCCGCCGTCGTCCACGGCAACGCCGACCG

		CGCGCTGCTGCAGGCGTACTCGATCGAACGACACGACGCGTGGGAGCAGAA<br>CGTCGCGAACGCCGGTAAGTCCACGCTCATCATGTCCCCGGGCACCCACGGC<br>TACCGCACCACCCGCGATGCCGTGCTCGCGCTCGCCGTGAACCGCCCCGAAT<br>TCGGGGAACTGATCAACCCGCGCCAGTCCAGCGCCACCCACGCCCGCCTGTC<br>GCCGCTGACCTGGCCGGTCGCGGAGGGCCTGACCGGGGTCCTGCCCGGCGAC<br>CCCGTCGAGGACCGCCGCGTGCGGGTCGCCACCGCCGGCGGCGGCACCGAG<br>TCGTCGCTCAACGCGGTGCGCGGCAACGGCTTCGCCGTCGTGGGCTTCGGTCT<br>CGACGCCGCCGGCAGCGCCGCGCTCGCGGCGGCCACGGCCGACCTCGCCGC<br>CGCGCTGGCGCCGGAGAGCGTGCGTGCCGTCGTCGTCGGTGCCGATAGTTCC<br>GGTGCCGACGGTTCCGATGATGTCACGGTGCTCGACGCGCCCGATCTGGCCG<br>AGGCGCTCGGCGCCACCCCGGGCGAGGCCTTCGTGATCCGGCCCGACGGGCT<br>GCTGCTGTGCCGGGTGCCGGTCGGCGCCCTGTCCGGTGTCGCCGCCGCACTCC<br>GCACCGGCACGGCCCCCGAGGGGCCGGCGGTCCCCGCGCGCCCGGCACAGA<br>TCCTCACAGAGGGCGAGCAGCGTCGTGAGAACGTCTGGCTCGGCCTGTCCGA<br>CGCGCTCGACCAGGTCGATCCGTCCGACCGGGAAGGATTTTTGGTGCGGCTC<br>GCGCTGCAGCTCGGGTCGCAGGCCGGTCACCGCGAGTTCGAAGAAGCCATCG<br>CAGCGGCGACCGACACGTCGCCGGTGTCCACCCGGGAACTGGCGCCACAGG<br>CCTGA
Rhodococcus ruber YC-YT1_cytochrome P450	WP_102031076.1	ATGAGCACTACAGAGCAGGTCACGGAGCTGATCCCGGACACCGTCGCCCGG<br>CAGATCGTGCTGCCCGAAGGGCACCGGGACAACGACGCGTTGTTCGAGGCCT<br>ACCGCTGGCTGCGTGAGAACAACCCGCTCGGCCGCGCCGTGGTCGAGGGATA<br>CGACCCGCTGTGGCTGGTGAGCAAGCACGCCGACATCATGGAAATCGAGCG<br>CCAGCCCGACATCTTCACCAGCGCAGGCGGCGACGACAAGGGGTCCGTCAA<br>CCCGATCCTGGCGAACCAGGCCGGCGACGAGTTCACCAAGAGCATCAACAA<br>CGGCAGTCTCCGCATCCTCGAGACCCTCACCTACCTCGATCCGCCGGAGCAC<br>ACCGAGATCAAGGACATCGCGATCGACTGGTTCCGGCCGACCAACCTCGCGA<br>AGTGGGAAACCGTCATCCGTGACCTTGCCAAGGCGTCCGTCACCCGGCTCCT<br>CGAGACGGACGGACGGATCGACTTCGTCAAGGACTTCGCCCTCCACTACCCG<br>CTGCACGTCATCATGAGCCTGTTCGGGGTGCCCGAGGAAGACGAACCCCGCA<br>TGATGGCCCTGACGCAAGAGTTCTTCGGGACGGCCGACCCCGATGCCGTCCG<br>TGAGGATGTCGAACCGCTCACCCCGGACGCCGCCGCCAAGCAGTGGGTGGCC<br>ACGATCCAGGACTTCTACGCGTACTTCGAACGGCTGCTCCAGGATCGCCGCG<br>CCAATCCTCGAGACGACCTCGCCACGATCATCTCCTCGGCCCGCGACAAGGA<br>CGGCGAGTACTACCCGAACGAGATGGCCTACGGTTGGTTCATCGCCATCGCG<br>ACCGCGGGCCACGACACCACCTCGAGCACCCTCGCGGGCGGCATGCTCGAG<br>CTCGGCCGCAACCCGGACCAGCTCGCCAAGGTGAAGGCCGACCTGGCGCTCG<br>CTCCGCACCTTGTGAACGAGTCGCTGCGCTGGGCCTCGCCGGTCAAGCACTTC<br>ATGCGTCAGGCGACGCAGGACTACACGCTGCGCGGCCGCGACATCAAGAAG<br>GGCGACCGCTTCATGCTGCTGTACCAGTCGGGCAACCGCGACGCGGAGGTCA<br>TCCCCGACCCCGATCGCTTCGACATCACCCGCCGGCCCAACAAGCACATCGC<br>CTTCGGCTACGGTCCGCACATGTGCATCGGCCAGCACCTGGCGAAACTGGAA<br>CTGCGCATCATGTTCGAAGAACTGCTCCCCCACATCGAGTCCCTCGCGCTGGT<br>GGACGACACGAAGATGATCCAGACGAACTTCGTGGGCGGGCTCAAGAACAT<br>GCCCGTCCAGATCGAGTTCACCGGGTAA
Rhodococcus ruber YC-YT1_methane	WP_010596327.1	TTGAGTAGGCAGAGCCTGACCAAGGCCCATGCAAAGATCACCGAGCTGACG<br>TGGGAACCGACGTTCGCGACGCCGGCCACCCGCTTCGGCACCGACTACACGT<br>TCGAGAAGGCCCCCAAGAAGGACCCTCTCAAGCAGATCATGCGGTCCTACTT<br>CCCCATGGAGGAGGAGAAGGACAACCGGGTCTACGGCGCGATGGACGGCGC

monooxygenase		GATCCGCGGCAACATGTTCCGCCAGGTCCAGCAGCGCTGGCTGGAGTGGCAG<br>AAGCTGTTCCTCTCGATCATCCCGTTCCCGGAGATCTCCGCGGCCCGCGCGAT<br>GCCGATGGCCATCGACGCGGTGCCCAACCCCGAGATCCACAACGGTCTCGCG<br>GTGCAGATGATCGACGAGGTCCGGCACTCGACGATCCAGATGAACCTCAAG<br>AAGCTCTACATGAACAACTACATCGATCCCGCCGGGTTCGACATGACCGAGA<br>AGGCGTTCGCGAACAACTACGCGGGCACCATCGGCCGGCAGTTCGGGGAGG<br>GCTTCATCACCGGTGACGCGATCACCGCGGCCAACATCTACCTGACCGTGGT<br>CGCGGAGACCGCCTTCACGAACACCCTGTTCGTGGCGATGCCCGACGAGGCC<br>GCCGCCAACGGTGACTACCTGCTGCCCACCGTGTTCCACTCGGTGCAGTCCGA<br>CGAGTCGCGCCACATCTCCAACGGCTACTCGATCCTGCTCATGGCCCTGGCCG<br>ACGAGCGGAACCGGCCGCTGCTCGAACGCGACCTGCGCTACGCCTGGTGGA<br>ACAACCACTGCGTGGTCGACGCCGCGATCGGCACGTTCATCGAATACGGCAC<br>CAAGGACCGCCGCAAGGACCGCGAGAGCTACGCCGAGATGTGGCGGCGGTG<br>GATCTACGACGACTACTACCGCAGCTACCTGCTCCCCCTCGAGAAGTACGGG<br>CTCACCATTCCGCACGATCTCGTCGAGGAGGCGTGGAAGCGCATCACCGAGA<br>AGGGTTACGTCCACGAGGTGGCCCGGTTCTTCGCCACGGGCTGGCCGGTGAA<br>CTACTGGCGGATCGACGCCATGACCGACGCGGACTTCGAGTGGTTCGAGGAG<br>AAGTACCCCGGCTGGTACTCCAAGTTCGGCAAGTGGTGGGAGAACTACAACC<br>GCCTCGCCTACCCCGGCCGCAACAAGCCGATCGCGTTCGAGGAAGTCGGATA<br>CCAGTACCCGCACCGCTGCTGGACCTGCATGGTGCCGGCCCTGGTCCGCGAG<br>GACATGGTCGTGGAGAAGGTCGACGACCAGTGGCGGACCTACTGCTCGGAG<br>ACGTGCTACTGGACCGACGCGGTCGCCTTCCGCGGTGAGTACGAGGGCCGGC<br>CCACGCCGAACATGGGCCGTCTCACCGGTTTCCGGGAATGGGAGACCCTGCA<br>CCACGACAAGGATCTCGCCGACATCGTGCAGGACCTCGGGTATGTGCGCGAC<br>GACGGCAAGACCCTCGTCGGCCAGCCGCACCTCGATCTCGACCCGAAGAAG<br>ATGTGGACCCTCGACGACGTGCGGGGCAACACCTTCCAGAGCCCGAACGTCC<br>TGCTGAACCAGATGACGGACGAGGAGCGCGCAGCGCACATCGCGGAGTACC<br>GCGCCGGCGCCACGCCGCTCTGA
Rhodococcus ruber NBRC15591_alkane hydroxylase	BAV60970.1	CGAGTTGGGTCATAAGAAGGACGACCTCGAACGGTGGCTGTCGAAGATCACC<br>CTGGCCCAGACCGGCTACGGCCACTTCTACATCGAACACAACCGCGGCCACC<br>ACGTGCGGGTCTCCACCCCGGAGGACCCGGCCTCGGCCCGTTTCGGCGAATC<br>GTTCTGGGCGTTCCTGCCGCGCAGCGTGTGGGGCAGCCTGAAGTCGTCGTGG<br>GAGCTCGAGAAGACCCGCCTGCAGCGACTCGGCAAGAGCCCGTGGACCCTG<br>CGCAACGACGTCCTCAACGCGTGGGCGATGTCGGTGGTCCTGTTCGGCGTGCT<br>CGTCGCGGTGTTCGGCCCGGTCATCGTTCCGTTCCTGATCCTGCAGGCCGTCT<br>ACGGTTTCTCCCTGCTCGAGACGGTGAACTACCTCGAGCACTACGGACTGCTG<br>CGGCAGAAGACGGCCTCGGGCCGGTACGAGCGCTGCACCCCCGCGCACAGC<br>TGGAATTCCGACCACATCGTCACCAACATCTTCCTGTACCACCTGCAGCGACA<br>C
